# *Bigger* versus *smaller*: Children's understanding of size comparison words becomes more precise with age

**DOI:** 10.1111/cdev.14182

**Published:** 2024-11-01

**Authors:** Alissa L. Ferry, Mia G. Corcoran, Emily Williams, Sheila M. Curtis, Cathryn J. Gale, Katherine E. Twomey

**Affiliations:** ^1^ Division of Psychology, Communication, and Human Neuroscience University of Manchester Manchester UK

## Abstract

The ability to compare plays a key role in how humans learn, but words that describe relations between objects, like comparisons, are difficult to learn. We examined how children learn size comparison words, and how their interpretations of these change across development. One‐hundred‐and‐forty children in England (36–107 months; 68 girls; majority White) were asked to build block structures that were *bigger*, *longer*, *smaller*, *shorter*, or *taller* than an experimenter's. Children were most successful with words that refer to size increases. Younger children were less accurate with *smaller* and *shorter*, often building bigger structures. The dimensional aspect of *taller* emerged gradually. These findings suggest that children's interpretation of the meaning of size comparison words changes and becomes more precise across development.

**Abbreviation**
OR: odds ratio

Comparison— the process of assessing the similarities and differences among objects, events, or concepts—plays a key role in how humans reason and learn (Gentner, 1983; Gentner et al., 2003). It affects how children learn language, identify categories, and reason analogically. While many studies have shown how children benefit from the process of comparing and how this changes as children develop, less work has investigated how children talk about comparisons and how the language used to describe comparisons develops. Here, we investigate how children learn the meaning of a specific subset of comparative words, size comparison words (e.g., *bigger*, *smaller*), and how their interpretation of these words changes across development.

The ability to make comparisons is a cornerstone of higher reasoning abilities and a powerful method for acquiring and reasoning about information (Gentner et al., 2003). Comparison facilitates the learning and retention of new information (Gentner et al., 2009; Kurtz & Loewenstein, 2007; Loewenstein & Gentner, 2005; Oakes et al., 2009; Richland et al., 2017), creativity in problem‐solving (e.g., Gentner et al., 2003; Gentner & Markman, 1997; Gick & Holyoak, 1980), and the acquisition of abstract rules and categories (Doumas & Hummel, 2013; Gentner & Medina, 1998; Gick & Holyoak, 1983; Kurtz et al., 2013). Learning outcomes are facilitated by comparison in academic settings such as science (Gentner et al., 2016; Jee et al., 2013) and mathematics (Rittle‐Johnson & Star, 2007, 2009). Comparison also facilitates the acquisition of various skills across development. Comparing multiple exemplars of objects or events improves children's ability to learn and remember labels paired with those exemplars (Childers, 2020; Childers et al., 2017; Gentner & Namy, 1999; Twomey et al., 2014), facilitates the acquisition of abstract concepts and categories (Anderson et al., 2018; Christie & Gentner, 2010; Ferry et al., 2015; Namy & Clepper, 2010; Namy & Gentner, 2002; Vukatana et al., 2015), and improves children's ability to reason about the social world (Christie, 2017; Hoyos et al., 2020). Thus, the ability to make comparisons is foundational to higher‐order reasoning and plays a key role in how humans think and learn, across a large number of domains.

Language allows us to talk about comparisons and can facilitate the comparison process. One way that language facilitates comparisons is through labels. Even for infants, pairing various exemplars with the same label invites comparison and highlights the commonalities between the exemplars, while pairing exemplars with different labels highlights the differences between them (Althaus & Westermann, 2016; Ferry et al., 2010; Plunkett et al., 2008; Waxman & Markow, 1995). However, words can also refer directly to relations between objects, such as spatial relations (e.g., *in*, *on*, *middle*), matching relations (e.g., *same*, *different*), quantity relations (e.g., *more*, *less*), and comparative relations (e.g., *bigger*, *smaller*; *faster*, *slower*). These relational words do not refer to easily individuated referents and are learned later in development than words that do refer to easily individuated objects (Gentner, 1982; Gleitman et al., 2005).

Previous work has shown a protracted development of relational language. By around 3 years of age, children show evidence that novel relational language facilitates comparison and increases attention to the relational structure (Christie & Gentner, 2010; Gentner et al., 2011). For example, one study (Christie & Gentner, 2014) tested 2½‐ to 4‐year‐olds on a relational‐match‐to‐sample task, in which participants match an identity relation (e.g., the standard AA should match BB instead of CD). While only the 4‐year‐olds initially showed above chance performance on the task, labeling the standard (“See this one? This is a truffet!”), improved performance, even in 2½‐year‐olds. Thus, even young children can link words to relational concepts. Other work has shown that known relational labels can improve children's task performance on relational tasks, indicating that there is some understanding of the meaning of the relational words. For example, 4‐year‐olds are better at finding a sticker in a corresponding box if relational language (e.g., *on the box*) is used in the training phase (Loewenstein & Gentner, 2005). However, other relational words emerge later and some words undergo changes, with some aspects of meaning being acquired before others. One study (Simms & Gentner, 2019) showed that a majority of 3‐year‐olds understood the meanings of *in*, *on*, *under* but not words like *middle* and *between*. While 5‐year‐olds generally understood the word *between*, most still struggled with the more precise *middle*. Moreover, their understanding of the words *middle* and *between* significantly predicted their performance on a task that involved finding a treasure box at the midpoint between two flags. Thus, relational language and the link between that language and relational concepts starts to emerge in children before the age of three, and continues to improve over several years.

We focus on a particular subset of relational language: words that describe size comparisons. Size comparison words can differ in their polarity (e.g., positive words like *bigger* highlight the increase in size, while negative words like *smaller* highlight the decrease), and in whether they refer to particular dimensions (e.g., *taller* refers to an increase in a specific dimension; *bigger* can be applied in any dimension). Thus, investigating the development of these words can clarify how children learn size comparison words in general, and whether differences in the specific nature of the relation influence the developmental trajectory. Previous work on size comparisons and on related words, such as size words (e.g., *big*), size superlatives (e.g., *biggest*), or quantifiers (e.g., *more*, *most*) shows that these words start to appear in children's speech starting around age 3 years, and that their frequency increases with age (Frausel et al., 2020). Our focus of interest, however, is on how children interpret the meanings of these words and how that changes across development. Research on children's understanding of the meanings of these words has shown mixed results, both in what children know and how this changes across age.

Some research suggests that children learn positive polarity words before negative polarity words (Barner & Snedeker, 2008; Donaldson & Wales, 1970; Silvey et al., 2017; Smith et al., 1988, though see Bishop & Bourne, 1985; Ravn & Gelman, 1984), potentially by several years (Pagliarini et al., 2022) and indeed that negative polarity words are often interpreted with a positive meaning. For example, 3‐year‐olds can identify that the word *more* refers to an increase in quantity (Odic et al., 2013), but at the same age often understand the word *less* to also mean an increase in quantity (Donaldson & Balfour, 1968), and it is not until several years later that *less* is reliably encoded as a decrease (Palermo, 1973). Similarly, 4‐year‐olds learn novel positive polarity determiners (semantically similar to *most*) more easily than negative ones (semantically similar to *fewest*) (He & Wellwood, 2022). Research has also looked at comparative words specifically. Some work shows that even children as young as 2‐ to 3‐year‐olds understand both positive and negative words like *big* and *little* (Smith & Sera, 1992) and that children as young as 2½ years can succeed on pointing tasks (e.g., “Show me the *X* that is bigger/smaller than the *Y*.”), but not production tasks (e.g., Layton & Stick, 1979), showing at least some understanding of simple positive and negative words. Other work (Bishop & Bourne, 1985) has argued that this success on the pointing task can be explained by children interpreting only the first part of the sentence (e.g., “Show me the truck that is big.”) without attending to the comparative aspect, though still showing some understanding of the roots. Other studies conclude that children may not have the same interpretations of comparatives as adults and that their interpretation of even positive comparatives changes across development (Bishop & Bourne, 1985; Gathercole, 1985; Syrett et al., 2010). Overall, studies of comparatives have tested a range of words, including words that vary in polarity, but these are generally not broken down by word or even polarity, making it difficult to draw conclusions about the trajectories of different types of words. A notable exception is Bishop and Bourne (1985), who found no effect of polarity in 4‐ to 7‐year‐olds. However, their participants were pre‐screened on the root words, and half of the 4‐year‐olds were excluded from the study based on pre‐test errors. While the pre‐test results were not described in detail, it is possible that errors were driven by negative polarity words, and participants who struggled with these words were excluded, which could account for the lack of polarity effects in the comparative word task. Thus, existing work suggests that size comparison words may be learned gradually and that different words may be learned on different trajectories, but this remains an open question.

Clark (1973, 2022) proposed a gradualist view of word meaning in language acquisition. This proposal suggests that children's initial representations of words may only partially capture the full meaning of the word and that meanings become more precise with development. This is particularly true with more abstract words. Children initially overextend color words and become more sensitive to the boundaries marked by those words with age (Wagner et al., 2013, 2018), initially misinterpret kinship words (e.g., interpreting *brother* as synonymous with *boy*; Haviland & Clark, 1974), and initially interpret *middle* as synonymous with *between*, before learning that *middle* has a more precise meaning (Simms & Gentner, 2019). The previous paragraph reviewed various evidence that positive polarity words might be learned before negative polarity words. Size comparison words also have an additional attribute, in that some words highlight specific dimensions (e.g., *bigger* vs. *taller*). These more precise definitions might also emerge gradually over time. Some work supports this view, with some evidence that 2‐ and 3‐year‐olds understand broader words like *big*, before more narrow words like *tall* and *long* (Tashiro, 1971). Other work suggests that children might attribute dimensional constraints to the word *big* at some points in development, interpreting the word too specifically. Three‐year‐olds have been shown to map *big* to the most salient dimension (Bausano & Jeffrey, 1975) and Maratsos (1973) showed that children's interpretations of the word *big* change between 3‐ and 5‐years, with younger children appropriately interpreting the word to include both height and width in a forced‐choice task and older children incorrectly interpreting the word to refer to the height dimension only. Layton and Stick (1979) showed in production tasks that 2.5‐ to 4.5‐year‐olds would swap positive and negative words (using *big* to refer to *small* and *small* to refer to *big*), particularly swapping in *big*, for negative polarity words, which they attributed to *big* serving as the most basic size reference. Thus, children's interpretations of the meanings of words may shift across development as they fine‐tune their word‐to‐meaning mappings, but it remains an open question whether this happens based solely on the development of an increased precision in meaning (as proposed by Clark, 1973, 2022) or whether polarity and dimensional attributes are added to broader words (and subsequently) pruned at a later point, as children refine the meanings of the words.

In sum, the previous work on comparative words has offered mixed results for how representations of these words develop in children. These mixed results may be due to differences in the tasks used, differences in age groups tested (including some studies with wide age ranges collapsed), differences in the words used (and collapsing analyses across different words), and unrelated task demands (e.g., complex syntax, memory demands). Our goal in the present study, then, was to determine how precisely children interpret the meaning of size comparison words and how that changes as they develop. To do this, we designed a block‐building task that worked equally well across a wide age range and allowed us to compare different words in the same participants. An experimenter built a baseline structure that was either four blocks long or four blocks tall. Four blocks were selected to allow structures to be easily built with more or fewer blocks. Long and tall baseline structures were used to capture any specifics related to dimensionality. Children were asked to build their own structure that was either *bigger*, *smaller*, *taller*, *shorter*, or *longer* than the baseline. This allowed us to see how children interpreted the meaning of each word. This design allowed more flexibility to demonstrate the interpretation than the forced‐choice tasks used previously (e.g., Bishop & Bourne, 1985; Layton & Stick, 1979), by allowing us to see how the child freely interpreted the word, both with the number of blocks used, and the dimensions that were changed from the baseline. The design also controls for key age‐related changes such as differences in syntactic processing (by using a simple question with the target word at the end) and memory demands (by keeping the baseline structure in view of the participants as they build). This allowed us to examine differences between the words, without interference from other changes in cognitive development. We selected these words because they are relatively common in children's input and include positive and negative polarity words and words with and without dimensional attributes. We coded trials for both overall accuracy and the type of structures built. To capture developmental changes in the perceived meanings, we tested children from 3‐ through 8‐years of age.

Based on previous work showing that the understanding of relational language shows a protracted development (e.g., Simms & Gentner, 2019), including some of the work with size words specifically (e.g., Bishop & Bourne, 1985; Gathercole, 1985; Syrett et al., 2010), we predicted that overall, children would improve on the task with age. We also predicted that the developmental trajectories would differ across words, in line with the gradualist view of word meaning. Specifically, if children gradually learn the different features of the words (Clark, 1973, 2022) and they begin with the semantic feature of a size change, we would expect that younger children would be more likely to initially treat all of the words as synonymous with *bigger*, increasing the size and without attention to specific dimensions. With age, we would expect children to add polarity as a feature of the words, distinguishing between words that note an increase or a decrease. Finally, we expected that the dimensional attributes (e.g., that *taller* refers to a change in the height dimension specifically) would emerge last.

## METHODS

### Participants

Participants were 204 children visiting the Manchester Museum (in Manchester, England). Families were approached if there was a child who appeared to be in the appropriate age range (3 years, 0 months to 8 years, 11 months). Of the participants, 74% were White, 6% were Asian, 3% were Black, 7% were mixed ethnicity, <2% were other ethnicities, and 8% opted not to answer. Of these, 140 were included in the final analysis and 64 were excluded for: refusing to participate after the caregiver signed the paperwork (5), experimenter error (1), missing demographics information (5), the presence of hearing or visual impairments or a developmental delay (19), or being multilingual (34). As our goal was to investigate the trajectory of the understanding of English words and it is not clear if multilingualism would impact that, we focus our analysis here on the monolingual children. An analysis including the multilingual participants is provided in the Supporting Information and shows globally similar patterns. The remaining participants were a mean of 5.5 years (range: 3 years, 0 months to 8 years, 11 months, see Figure S1) and included 72 boys (51%) and 68 girls (49%). Parents provided informed written consent and children gave verbal assent. Data were collected from August 2018 to March 2020.

### Materials and procedure

The experiment took place either in a study room separate from the museum exhibits or in a dedicated space in the museum cafe. A table and chairs were arranged for the child, caregiver, and an experimenter to sit on one side across from Experimenter 1. A set of 24 wooden blocks of varying colors with painted numbers and letters were the stimuli. Experimenter 1 had a sheet detailing the order of the trials hidden out of view of the participant. Experimenter 2 had a coding sheet to record what the child built, the responses to the questions, and the comments. The caregivers were allowed to sit next to the child or stand behind them. Caregivers were asked not to help or interfere. Experimenter 1 sat across from the participants and engaged with them in the block‐building task. Experimenter 2 stood or sat slightly behind the child and recorded the responses and relevant information about the trials (e.g., parental interference). Experimenter 1 introduced herself (and Experimenter 2) and explained the task. Children were told that in this game, the experimenter would build something with the blocks and then ask the child to build something and her friend (Experimenter 2) would write down what the child did. Once children agreed to participate, the study began.

**FIGURE 1:**
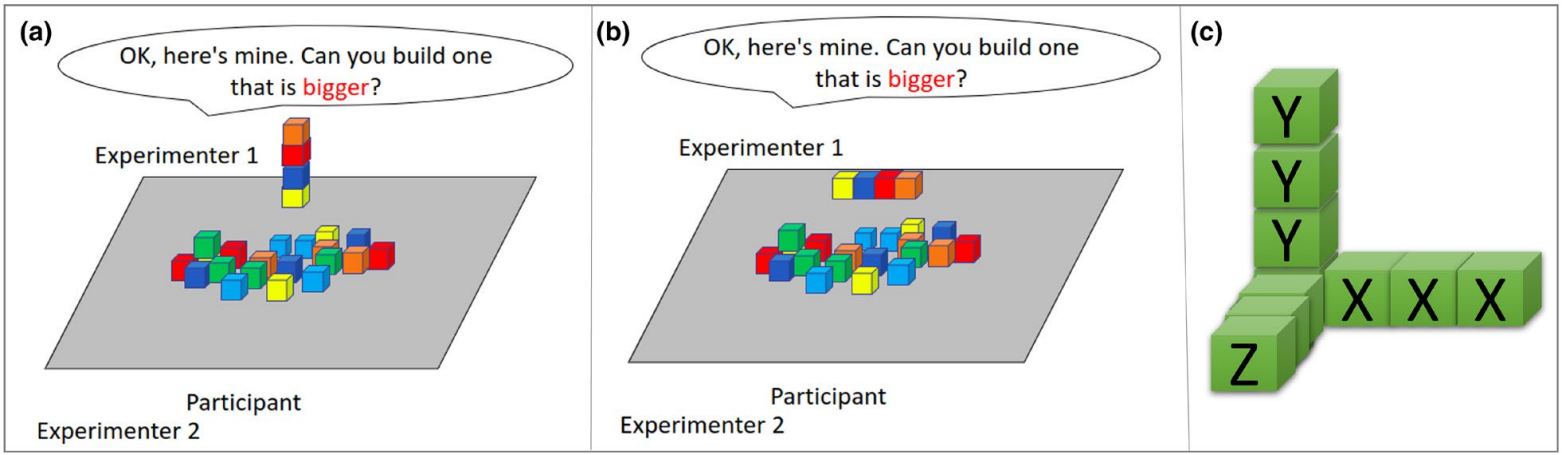
The experimental design of the study. Two experimenters sat at a table with a participant and 24 blocks on the table. On each trial, Experimenter 1 would build a structure with four blocks and ask the participants to build their own structure that was bigger, smaller, taller, shorter, or longer. Experimenter 2 recorded the structure built by the participant. (a) An example trial where Experimenter 1 built a Long baseline structure. (b) An example trial where Experimenter 1 built a Tall baseline structure. (c) Participant trial structures were coded using the number of blocks in the X‐, Y‐, and Z‐dimensions.

The study consisted of 10 trials. Sample trails are depicted in Figure 1a,b. On each trial, the experimenter would build a structure that was either four blocks touching in a row flat on the table (Long baseline) or four blocks stacked on top of each other (Tall baseline). Experimenter 1 would build her structure and say, “This one is mine. Can you make me one that is [target word]?” There were five target words: *bigger*, *longer*, *smaller*, *shorter*, and *taller*. Each word was presented once with the Tall baseline and once with the Long baseline. The order was determined by 10 randomization lists that were randomly assigned. The experimenters were trained to say all target words and carrier phrases with the same intonation and with their hands under the table to avoid potentially giving clues about the target word's meaning. The remaining 20 blocks were on the table between Experimenter 1 and the participant. The participant was free to use as many blocks as they wished and could freely build any structure. Experimenter 1 waited until the participant finished building and confirmed that they were done. Experimenter 2 recorded the structure (via drawings and text explanations). Experimenter 1 then asked, “Which one is [target word]?” and when the participant answered (e.g., “Mine,” “This one,” pointing gesture to one of the structures), Experimenter 1 confirmed the answer (e.g., “yours?,” “that one?,” pointing to the one the child pointed to) and waited for the child to confirm. Experimenter 2 recorded the responses. Experimenter 1 then asked the child to knock down the structures, mix up the blocks, and try another one. The study went on for a maximum of 10 trials or until the child no longer wanted to play. At the end, children were thanked and offered stickers. The University of Manchester ethical committee approved the study [#2018‐4815‐6565]. The study was not preregistered.

### Analysis plan

The data from paper coding sheets were input into a spreadsheet by trained coders. This included demographic information, trial information (e.g., trial number, word used, experiment structure), and the structures participants built and their responses to the experimenter's questions. The participant structures were drawn or explained on the sheet, and we quantified them with the total number of blocks, and the maximum number of blocks in the X, Y, and Z‐dimensions. The dimensions were defined with respect to the participant and are depicted in Figure 1c. The X‐dimension was the axis parallel to the tabletop and the participant (e.g., four blocks lined up in a row from left to right is four blocks in the X‐dimension). The Y‐dimension was defined as height (e.g., four blocks stacked on top of each other in a column is four blocks in the Y‐dimension). The Z‐dimension was defined as parallel to the tabletop, but perpendicular to the X‐dimension along the table (e.g., four blocks lined up in a flat row going away from the participant is four blocks in the Z‐dimension). Because we used comparative words, their meaning is defined with respect to a baseline reference (i.e., the structure built by Experimenter 1). Thus, we also described the participant's structure with reference to how each dimension differed from that baseline structure (i.e., whether the participant's structure decreased, did not change, or increased in each dimension). The initial baseline structure was either Long (four blocks lined up in a row; 4 blocks in the X‐dimension, 1 block in the Y‐dimension, and 1 block in the Z‐dimension) or Tall (1 block in the X‐dimension, 4 blocks in the Y‐dimension, and 1 block in the Z‐dimension; see Figure 1). Most trials (89% of the final dataset) consisted of shapes that were easy to define in terms of the number of blocks in each dimension (e.g., a series of blocks lined up in a horizontal row; a series of blocks stacked in a single column). In the remaining cases, children built more creative structures (e.g., a row of blocks horizontal with an extra column of more vertical blocks at the end; pyramids). Each trial was coded independently by at least two research assistants to ensure proper data entry and agreements of interpretation of the number of blocks in each dimension. Trials where there was uncertainty about the structure were excluded.

Prior to analysis, a total of 28 individual trials were excluded for various reasons: seven because the child was distracted during the trial, one for errors in the coding by the experimenter, two because of uncertainty about the drawing of the structure, four for being duplicate trials (i.e., experimenter error resulted in a condition being run twice, the second was excluded), and 14 for parent or sibling interference. In the final dataset, participants contributed 1304 total trials, an average of 9.31 trials per participant (range: 1–10). Trials were equally distributed across the 10 different conditions with an average of 130 trials per condition (range: 126–136).

#### Accuracy analysis plan

First, we analyzed children's responses to determine if children built the correct structures. Responses were coded as correct based on different parameters for each word and with respect to the experimenter's baseline structure. Note that there are potentially other ways of interpreting these words and that detailed analyses of the specific structures are provided in the next analysis. The word *bigger* was coded as correct if the structure used more blocks overall. The word *smaller* was coded as correct if the structure used fewer blocks overall. The root *long* has various interpretations (Oxford University Press, n.d.) that include both reference to a specific dimension (i.e., specifically referring to the X‐dimension) and in the context of other dimensions including the Y‐dimension (e.g., a long neck on a giraffe; long hair). Thus, the word *longer* was coded as correct if the structure increased in any dimension. The word *shorter* was coded as correct if the structure decreased in any dimension. The word *taller* was coded as correct if the structure increased specifically in the Y‐dimension.

The analysis was conducted using binomial generalized linear mixed‐effects models in the *lme4* package in R (Bates et al., 2015). The outcome variable was accuracy (1 = correct, 0 = incorrect), and random intercepts were set for each participant as participants completed multiple trials. Three models were compared: a baseline model with no fixed effects, a model with Age (as a continuous variable, centered so that 0 represented the youngest point in our time window, 3 years old, to ease interpretation), and Word (as a categorical variable, with *bigger* as the reference category) as fixed effects, and a model with their interaction. The best model was identified by the likelihood ratio test for model comparisons (Pinheiro & Bates, 2000). We used the R package *lmerTest* (Kuznetsova et al., 2017) to calculate *p*‐values. Raw model estimates are on the log‐odds scale and are presented here as odds ratios (ORs) to ease interpretation. ORs greater than 1 indicate a greater likelihood of a correct response compared with an incorrect response, less than 1 indicating a greater likelihood of an incorrect response, and ORs around 1 indicating an equal likelihood. Age coefficients greater than 1 indicate that accuracy increases with age and larger coefficients indicate a steeper slope (faster increase in accuracy). Post hoc tests of the estimated marginal means of the intercepts and age effects for each word were done using the *emmeans* R package (Lenth et al., 2019), with Holm‐Bonferroni adjustments.

#### Condition structure analysis

Overall accuracy measures provide an overview of whether children understand the meaning of comparative words consistent with adult interpretations. However, they offer less insight into what children might think these words mean if they respond incorrectly. For example, if children are incorrect on negative polarity words like smaller, are they building structures randomly, with no identifiable patterns? Or, if they understand that the word refers to a size comparison but have not identified that it is a negative differential in size (Clark, 1973, 2022), they might instead show a tendency to build larger structures. Thus, we modeled the particular structures that were built in each of the 10 different conditions to identify specific patterns in the structures and patterns that changed with age. We were interested specifically in the general patterns of the structures (e.g., whether structures decreased, increased, or stayed the same in a particular dimension, with respect to the baseline structure), rather than in how many blocks were used in a particular dimension (e.g., a five‐block tall structure and a 10‐block tall structure would both meet the definition of a bigger structure). Therefore, we coded the changes in each dimension (with respect to the baseline) as ordinal variables. If the experimenter's baseline structure was Long (four blocks in the X‐dimension, one in the Y‐dimension, and one in the Z‐dimension), the participant could build a structure that decreased, increased, or did not change in the X‐dimension, and increased or did not change in the Y‐ and Z‐dimensions. If the experimenter's baseline structure was Tall (four blocks in the Y‐dimension, one in the X‐dimension, and one in the Z‐dimension), the participant could build a structure that decreased, increased, or did not change in the Y‐dimension, and increased or did not change in the X‐ and Z‐dimensions. Thus, the ordinal ranking was a decrease, no change, or an increase for dimensions in which the baseline structure was four blocks, and no change, or an increase for dimensions in which the baseline structure was one block. Because it was possible to change multiple dimensions at once, we opted to use multivariate ordinal models. We also suspected that changes in the various dimensions would not be independent. That is, that certain patterns of X and Y changes might exist. Thus, we opted to use multivariate ordinal models that account for this potential correlation between the different variables, using the *mvord* R package (Hirk et al., 2020). Changes in the Z‐dimension were rare (35 trials, 2.7% of trials), so we only included X and Y measures in the models. In each condition, we built one model with only the ordinal response measure (the base model) and one model with the addition of Age as a continuous predictor. Age was centered so that 0 represented the youngest point in our time window (3 years old). Models were compared using a likelihood ratio test in the *lrtest* R package (Zeileis & Hothorn, 2002). Conditions were analyzed separately to identify particular developmental changes for the structures in each word, and because different experimenter baseline structures offered different constraints on the thresholds (i.e., if the baseline structure was long, the Y‐dimension could either not change or increase; if the baseline structure was tall, the Y‐dimension could either decrease, not change or increase). Because we compared across 10 conditions, we used a stricter threshold (*p*‐value < .005) to determine the significance of the model comparison tests. However, the interpretation of the *p*‐values for model coefficients was referenced to the .05 threshold.

#### Multivariate ordinal model interpretation

As in the binomial model, the coefficients are presented as ORs. There are several key measures in the results. First, within each model are threshold coefficients, which explain the likelihood of different outcomes in the structures in the X‐ and Y‐dimensions. Because the outcomes are ordinal (decrease|no change|increase, in any particular dimension), the thresholds examine the odds of the changes in a particular dimension at each side of a cut point (e.g., the odds of decrease compared with no change or increase; the odds of decrease or no change compared with increase). For measures with three options (decrease, no change, increase) there are two cut points (decrease|nochange; nochange|increase) and for measures with two options (no change, increase) there is one cut point (nochange|increase). Coefficients greater than 1 indicate that options to the left of the cut point are more likely than those to the right. For example, a threshold value of 2.0 would indicate that values to the left are twice as likely (e.g., when examining the nochange|increase cut point for the X‐dimension for a baseline structure that was tall, a threshold coefficient of 2.0 would mean that children were twice as likely to not change in that X‐dimension as they were to increase). Coefficients less than 1 indicate that the option to the left of the cut point is less likely than the option to the right. For example, a threshold value of .5 would indicate that values to the left are half as likely (e.g., when examining the nochange|increase cut point for the X‐dimension in a baseline structure that was tall, a threshold coefficient of .5 would mean that children were half as likely to not change in that X‐dimension as they were to increase in the X‐dimension). Coefficients around 1 indicate equal likelihood of a response being on each side of a cut point (e.g., a threshold coefficient of 1 in the X‐dimension for a baseline structure that was tall would mean that children were equally as likely to not change in that X‐dimension as they were to increase).

More details about the interpretation of these models are available in the Supporting Information. In the base model, the threshold coefficients represent the likelihood of the whole dataset. If Age is included, the threshold coefficients represent the estimated intercept (threshold coefficient for 3‐year‐olds) and the Age coefficients represent the change in the OR of moving up on the ordinal scale for each year on the age scale. Values above 1 indicate that with age, participants move in the direction of increasing in that dimension while values less than 1 indicate that they become less likely to increase in that dimension, and values around 1 indicate no change in that dimension with age. Finally, the correlated error structure between X‐ and Y‐dimensions explains the correlation between the dimensional measures. Positive correlations indicate that structures that move up the ordinal scale in one dimension are more likely to also move up the ordinal scale in the other dimension. Negative correlations indicate that structures that move up the ordinal scale in one dimension are more likely to also move down the ordinal scale in the other dimension. High positive correlations would be expected if, for example, participants tend to increase structures in both directions. High negative correlations would be expected if, for example, participants tend to increase structures in a specific dimension and not change or decrease in the other dimension. No correlations would be expected if, for example, participants show mixed responses in one dimension and a consistent response in another dimension.

## RESULTS

Data and data processing information are available on the Open Science Framework (https://osf.io/ekvqc/).

### Accuracy results

**FIGURE 2:**
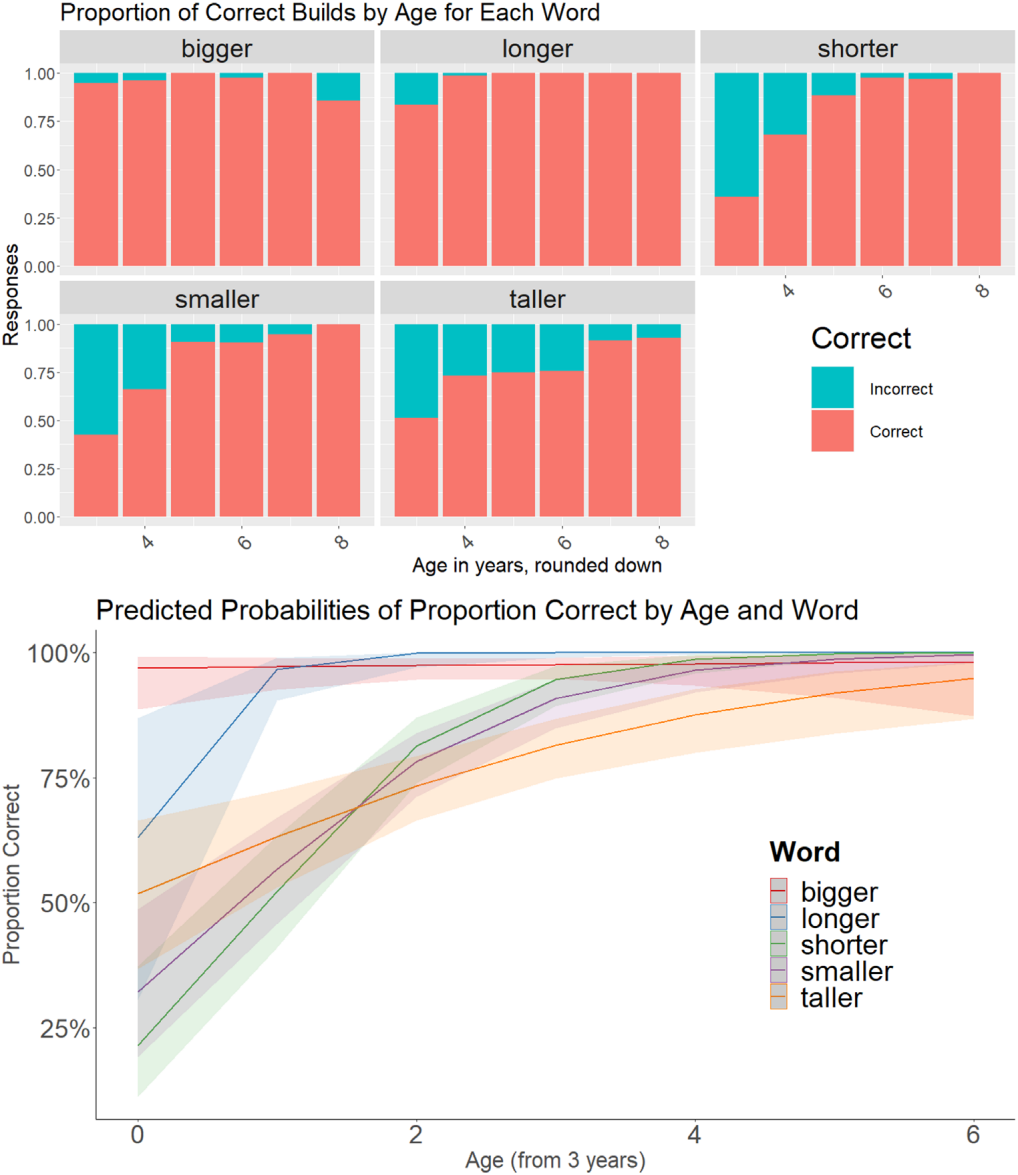
The accuracy for each word across the age range. (Top) The proportions of correct and incorrect trials for each word, binned by age (rounded down). (Bottom) The model predicted probabilities of building accuracy for each word, across the age range.

The model with the main effects of Age and Word was a better fit than the baseline model (*p* < .0001) and the model with the interaction of Age and Word was an even better fit (*p* < .0001). The models are available in Table 1. The predicted accuracy for the Age and Word model is in Figure 2. Because there was an interaction of Age and Word, we compared the estimated marginal means at the start of the age window (3‐year‐olds) to assess the initial accuracy for each word and the slopes to assess how the accuracy changed with age. Three‐year‐old children were more likely to be correct for *bigger* (OR = 31.39, *p* < .0001), followed by *longer* (OR = 1.71, *p* = .88), *taller* (OR = 1.07, *p* = .88), and *smaller* (OR = .48, *p* = .11), where responses were mixed, and then *shorter* (OR = .28, *p* < .01) where responses were more likely to be incorrect. The intercept for *bigger* differed from all other words, and the other words did not differ from each other, except for a significant difference between *taller* and *shorter*.

**TABLE 1:** Summary of the binomial generalized linear mixed‐effects models for the trial accuracy.

Predictors	Null model (NullModel_Accuracy)			Age and word main effects (Age_Word_Model_Accuracy)			Age and word interaction (Age_WordModel_Accuracy)		
	Odds ratios	CI	*p*	Odds ratios	CI	*p*	Odds ratios	CI	*p*
(Intercept)	7.44	5.66–9.79	**<.001**	8.36	3.76–18.59	**<.001**	31.39	7.79–126.38	**<.001**
Word [longer]				1.07	0.38–2.97	.901	0.05	0.01–0.37	**.003**
Word [shorter]				0.08	0.04–0.18	**<.001**	0.01	0.00–0.04	**<.001**
Word [smaller]				0.08	0.04–0.18	**<.001**	0.02	0.00–0.07	**<.001**
Word [taller]				0.06	0.03–0.14	**<.001**	0.03	0.01–0.15	**<.001**
Age centered				2.27	1.89–2.73	**<.001**	1.08	0.65–1.79	.765
Word [longer] × age centered							15.44	2.36–100.78	**.004**
Word [shorter] × age centered							3.68	1.92–7.09	**<.001**
Word [smaller] × age centered							2.54	1.39–4.63	**.002**
Word [taller] × age centered							1.48	0.86–2.57	.161
Random effects									
*σ* ^2^	3.29			3.29			3.29		
*τ*_00_	1.11_ParticipantID_			0.43_ParticipantID_			0.45_ParticipantID_		
ICC	.25			.11			.12		
*N*	140_ParticipantID_			140_ParticipantID_			140_ParticipantID_		
Observations	1304			1304			1304		
Marginal *R* ^2^/conditional *R* ^2^	.000/.252			.449/.512			.720/.754		
Akaike information criterion	1078.391			870.644			845.580		

The bold denotes the major conditions and significance tests results of the models.

We evaluated the model estimates for the slopes for each word across the age range. For *bigger*, there was no change across the age range (OR = 1.08, *p* = .77). The other four words showed significant positive slopes. *Longer* showed the steepest slope (OR = 16.7, *p* = .0046), followed by *shorter* (OR = 3.98, *p* < .0001), and *smaller* (OR = 2.74, *p* < .0001). Finally, taller showed a positive slope, but shallower (OR = 1.60, *p* = .0005). The slope for *bigger* was significantly different from all other words, except *taller*. No other slopes differed.

In sum, 3‐year‐olds show different accuracy for the different words and are most accurate with *bigger*, with lower accuracy for the other four words. Across the age range, children show no changes in their accuracy for *bigger*, but they do show improvements in accuracy in the other four words.

### Condition structure analysis results

**FIGURE 3:**
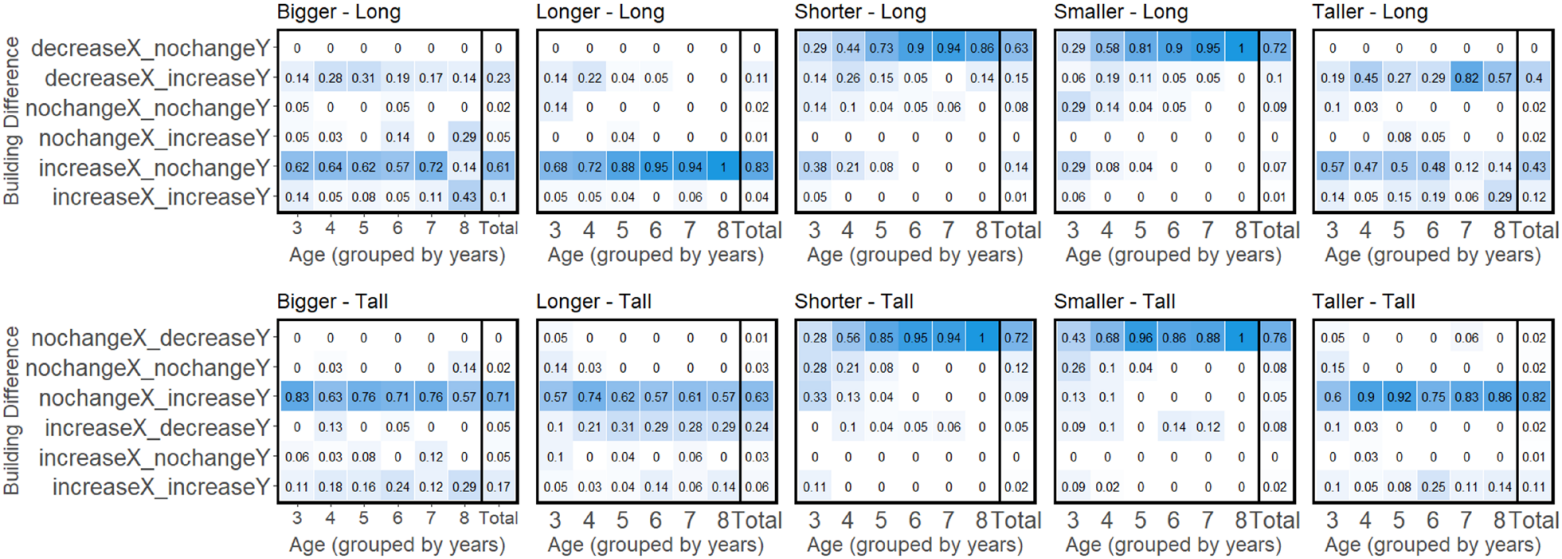
Heat maps of each of the 10 conditions. The proportion of structures for each age bin (age in years, rounded down). Each plot shows the proportion of the types of structures participants made, in terms of the ordinal changes (decreasing, increasing, or not changing in a particular dimension). Baseline long structures are plotted on the top and baseline tall structures are plotted on the bottom.

For each condition, we compared models with and without Age. Model summaries are in Table 2. In addition, heat maps of the structures with respect to the baseline across binned (rounded down) age groups are in Figure 3. The best model and a summary of the common patterns across the group is described below.

**TABLE 2:** Summary of the multivariate ordinal regression models for structures built in each condition.

Model comparisons for each word in the “long” condition										
	Bigger		Longer		Shorter		Smaller		Taller	
	Null model	Age model	Null model	Age model	Null model	Age model	Null model	Age model	Null model	Age model
X decreaseX|nochangeX	0.484**** [0.379, 0.618]	0.488** [0.307, 0.774]	0.262**** [0.179, 0.384]	0.706 [0.334, 1.493]	2.569**** [1.977, 3.338]	0.629 [0.349, 1.132]	2.16**** [1.693, 2.756]	0.663 [0.398, 1.105]	0.774* [0.614, 0.975]	0.494** [0.309, 0.790]
X nochangeX|increaseX	0.613**** [0.487, 0.771]	0.601* [0.378, 0.956]	0.332**** [0.253, 0.434]	0.863 [0.458, 1.625]	4.112**** [2.958, 5.716]	1.167 [0.645, 2.114]	2.884**** [2.202, 3.778]	0.959 [0.599, 1.537]	0.857 [0.691, 1.064]	0.558* [0.357, 0.872]
Y nochangeY|increaseY	1.297* [1.035, 1.624]	1.604* [1.024, 2.512]	2.914**** [2.135, 3.977]	1.45 [0.766, 2.742]	3.405**** [2.535, 4.573]	2.188* [1.153, 4.152]	2.787**** [2.137, 3.635]	1.735* [1.080, 2.789]	0.895 [0.713, 1.124]	1.764* [1.092, 2.851]
Corr X Y	−0.925**** [−1.034, −0.816]	−0.925**** [−1.034, −0.816]	−0.925**** [−1.085, −0.765]	−0.925**** [−1.151, −0.699]	−0.221 [−0.638, 0.196]	−0.404 [−0.847, 0.038]	−0.433* [−0.778, −0.088]	−0.63**** [−0.938, −0.323]	−0.925**** [−1.042, −0.808]	−0.925**** [−1.071, −0.779]
Age (X)		1.016 [0.859, 1.200]		1.607** [1.146, 2.255]		0.487**** [0.368, 0.644]		0.559**** [0.434, 0.719]		0.833* [0.712, 0.975]
Age (Y)		1.072 [0.915, 1.256]		0.753* [0.578, 0.983]		0.829 [0.641, 1.071]		0.808* [0.672, 0.970]		1.342** [1.126, 1.601]

Likelihood ratio test	n.s.		*p* < .001		*p* < .00001		*p* < .00001		*p* < .001	
Num.Obs.	132	132	132	132	127	127	131	131	130	130
Akaike information criterion (AIC)	297.97	299.82	184.14	172.4	242.88	210.54	291.46	255.43	316.44	306.46
Bayesian information criterion (BIC)	309.86	317.94	196.03	190.52	254.63	228.45	303.32	273.51	328.28	324.5

Model comparisions for each word in the “tall” condition										
	Bigger		Longer		Shorter		Smaller		Taller	
	Null model	Age model	Null model	Age model	Null model	Age model	Null model	Age model	Null model	Age model
X nochangeX|increaseX	1.842**** [1.451, 2.340]	1.979* [1.174, 3.338]	1.549*** [1.232, 1.948]	2.135** [1.332, 3.423]	3.542**** [2.640, 4.753]	2.884*** [1.655, 5.026]	4.364**** [3.116, 6.113]	3.124*** [1.632, 5.979]	2.978**** [2.266, 3.914]	3.281**** [1.862, 5.782]
Y decreaseY|nochangeY	0.188**** [0.128, 0.278]	0.205**** [0.114, 0.369]	0.482**** [0.380, 0.613]	0.45** [0.280, 0.724]	2.757**** [2.129, 3.570]	0.668 [0.365, 1.223]	2.116**** [1.657, 2.702]	0.354** [0.188, 0.668]	0.17**** [0.113, 0.256]	0.338** [0.153, 0.747]
Y nochangeY|increaseY	0.297**** [0.222, 0.398]	0.325*** [0.173, 0.611]	0.605**** [0.483, 0.759]	0.568* [0.364, 0.886]	4.256**** [3.061, 5.919]	1.196 [0.646, 2.214]	3.419**** [2.540, 4.604]	0.715 [0.378, 1.353]	0.227**** [0.164, 0.316]	0.464* [0.242, 0.888]
Corr X Y	−0.808**** [−1.008, −0.607]	−0.802**** [−1.019, −0.585]	−0.925**** [−1.005, −0.845]	−0.925**** [−1.047, −0.803]	0.209 [−0.174, 0.592]	0.147 [−0.320, 0.613]	0.072 [−0.338, 0.481]	−0.095 [−0.617, 0.428]	−0.524** [−0.887, −0.162]	−0.578** [−0.978, −0.178]
Age (X)		1.028 [0.856, 1.234]		1.13 [0.961, 1.327]		0.914 [0.743, 1.124]		0.864 [0.672, 1.111]		1.038 [0.855, 1.261]
Age (Y)		1.036 [0.821, 1.306]		0.979 [0.837, 1.144]		0.43**** [0.285, 0.649]		0.386**** [0.272, 0.547]		1.452* [1.049, 2.009]

Likelihood ratio test (*p*‐value)	n.s.		n.s.		*p* < .00001		*p* < .00001		n.s.	
Num.Obs.	126	126	131	131	136	136	128	128	131	131
Akaike information criterion	236.06	239.95	285.83	284.63	243.46	212.86	250.44	203.51	184.83	179.87
Bayesian information criterion	247.77	257.82	297.69	302.71	255.46	231.14	262.22	221.46	196.69	197.95

*Note*: Each condition has a null model with no fixed effects and one with age as a fixed effect. The coefficients are odds ratios (95% CIs in square brackets). The likelihood ratio test shows whether Age significantly improved the model fit. The conditions with a Long baseline are on the top and the conditions with the Tall baseline are on the bottom.

* *p* < .05;

** *p* < .01;

*** *p* < .001;

**** *p* < .0001.

#### Bigger—Long baseline

Age did not significantly improve the null model and did not influence either dimension. The null model showed that participants were significantly more likely to increase the X‐dimension and not change the Y‐dimension. Compared with the baseline structure (a row four blocks long) most participants (61%) increased in the X‐dimension only (e.g., a row of five or more blocks long) but some (23%) decreased in the X‐dimension and increased in the Y‐dimension (e.g., a single column two or more blocks high) and this did not differ by age.

#### Bigger—Tall baseline

Age did not significantly improve the null model and did not influence either dimension. The null model showed that participants were more likely to not change the X‐dimension and to increase the Y‐dimension. Compared with the baseline structure (a column four blocks tall), most participants (71%) increased in the Y‐dimension only (e.g., a single column of five or more blocks high) but (17%) increased in both dimensions (e.g., two adjacent columns each five blocks high) and this did not differ by age.

#### Longer—Long baseline

Age did significantly improve the null model (*p* < .001). At 3 years, there was no consistent pattern of changes in the X‐dimension, nor was there a consistent pattern in the Y‐dimension. There was a significant effect of Age in the X‐dimension, with increases in the X‐dimension becoming more likely with age and increases in the Y‐dimension becoming less likely with age. Overall, participants across the age range tended to build structures that increased in the X‐dimension with no change in the Y‐dimension (83%). Some participants built structures that decreased in the X‐dimension and increased in the Y‐dimension (11%) or that did not differ from the baseline (2%) and these participants were clustered at the lower ends of the age range. In sum, compared with the baseline structure (a row four blocks long), younger participants showed a range of responses, primarily increasing in the X‐dimension only (e.g., a row five or more blocks long) but some would decrease in the X‐dimension and increase in the Y‐dimension (e.g., a single column two or more blocks high). With age, participants were more likely to only increase in the X‐dimension.

#### Longer—Tall baseline

Age did not significantly improve the null model and did not influence either dimension. The null model showed that participants were significantly more likely to not change the X‐dimension and to increase the Y‐dimension. Compared with the baseline structure (a column four blocks tall), most participants (63%) increased in the Y‐dimension only (e.g., build a single column of five or more blocks) but some (24%) would decrease in the Y‐dimension and increase in the X‐dimension (e.g., a row five blocks long) and this did not differ by age.

#### Shorter—Long baseline

Age did significantly improve the null model (*p* < .0001). At 3 years, there was no consistent pattern of changes in the X‐dimension, but there was a consistent pattern of not changing in the Y‐dimension. There was a significant effect of Age in the X‐dimension, with decreases in the X‐dimension becoming more likely with age, and an effect of age in the Y‐dimension, with increases in the Y‐dimension becoming less likely with age. Overall, participants across the age range tended to build structures that decreased in the X‐dimension with no change in the Y‐dimension (63%), but some participants built structures that increased in the X‐dimension with no change to the Y‐dimension (14%) or that did not differ from the experimenter's structure (8%) and these participants were clustered at the lower ends of the age range. In sum, compared with the baseline (a row four blocks long), younger participants showed a range of responses, with some increasing in the X‐dimension only (e.g., a row five or more blocks long), some decreasing in the X‐dimension (e.g., a row three or fewer blocks long), and some building a structure identical to the experimenter's. With age, participants became significantly more likely to decrease in the X‐dimension (e.g., a row three or fewer blocks long).

#### Shorter—Tall baseline

Age did significantly improve the null model (*p* < .00001). At 3 years, there was a consistent pattern to not increase in the X‐dimension, and there was a consistent pattern to increase or not change in the Y‐dimension. There was no significant effect of Age in the X‐dimension but there was a significant effect of Age in the Y‐dimension, with decreases in the Y‐dimension becoming more likely with age. Overall, participants across the age range tended to build structures that decreased in the Y‐dimension with no change in the X‐dimension (72%), but some participants built structures that increased in the Y‐dimension with no change in the X‐dimension (9%), or that did not differ from the experimenter's structure (12%) and these participants were clustered at the lower ends of the age range. In sum, compared with the baseline (a column four blocks tall), younger participants showed a range of responses, with some participants increasing in the Y‐dimension only (e.g., a column five or more blocks tall), some decreasing in the Y‐dimension only (e.g., a column three or fewer blocks tall) and some building a structure identical to the experimenter's. With age, participants became much more likely to decrease in the Y‐dimension only (e.g., a column three or fewer blocks tall).

#### Smaller—Long baseline

Age did significantly improve the null model (*p* < .0001). At 3 years, there was no consistent pattern of changes in the X‐dimension, but there was a consistent pattern of not changing in the Y‐dimension. There was a significant effect of Age in the X‐dimension, with decreases in the X‐dimension becoming more likely with age, and no effect of age in the Y‐dimension. Overall, participants across the age range tended to build structures that decreased in the X‐dimension with no change in the Y‐dimension (72%), but some participants built structures that increased in the X‐dimension with no change to the Y‐dimension (7%) or that did not differ from the experimenter's structure (9%) and these participants were clustered at the lower ends of the age range. In sum, compared with the baseline (a row four blocks long), younger participants showed a range of responses, with some increasing in the X‐dimension only (e.g., a row five or more blocks long), some decreasing in the X‐dimension (e.g., a row three or fewer blocks long), and some building a structure identical to the experimenter's. With age, participants became more likely to decrease in the X‐dimension (e.g., a row three or fewer blocks long).

#### Smaller—Tall baseline

Age did significantly improve the null model (*p* < .00001). At 3 years, there was a consistent pattern to not increase in the X‐dimension, but no consistent pattern in the Y‐dimension. There was no effect of Age in the X‐dimension but there was a significant effect of Age in the Y‐dimension, with decreases in the Y‐dimension becoming more likely with age. Overall, participants across the age range tended to build structures that decreased in the Y‐dimension with no change in the X‐dimension (76%), but some participants built structures that increased in the Y‐dimension with no change in the X‐dimension (5%), or that did not differ from the experimenter's structure (8%) and these participants were clustered at the lower ends of the age range. In sum, compared with the baseline structure (a column four blocks tall), younger participants showed a range of responses, with some participants increasing in the Y‐dimension only (e.g., a column five or more blocks tall), some decreasing in the Y‐dimension only (e.g., a column three or fewer blocks tall) and some building a structure identical to the experimenter's. With age, participants became much more likely to decrease in the Y‐dimension only (e.g., a column three or fewer blocks tall).

#### Taller—Long baseline

Age did improve the null model (*p* < .001). At 3 years, there was a consistent pattern of increasing in the X‐dimension, and not changing in the Y‐dimension. There was a significant effect of Age in the X‐dimension, with increases in the X‐dimension becoming less likely with age, and a significant effect of age in the Y‐dimension, with increases in the Y‐dimension becoming more likely with age. Overall, participants across the age range tended to build structures that increased in the X‐dimension with no change in the Y‐dimension (43%) or structures that increased in the Y‐dimension and decreased in the X‐dimension (40%), with some participants increasing in both dimensions (12%). Increases in the Y‐dimension were predominantly at the higher ends of the age range. In sum, compared with the baseline (a row four blocks long), younger participants tended to increase in the X‐dimension only (e.g., a row five or more blocks long) though some showed increases in the Y‐dimension, either while decreasing the X‐dimension (e.g., a single column of two or more blocks), or increasing both dimensions (e.g., two five‐block rows stacked on each other) and others building a structure identical to the experimenter's. With age, participants became less likely to increase in the X‐dimension and more likely to increase in the Y‐dimension (e.g., a single column of two or more blocks; two four block rows stacked on top of each other).

#### Taller—Tall baseline

The model comparisons showed that Age marginally improved the null model but was not significant after correcting for multiple comparisons (*p* > .005). Age did not influence the structures in the X‐dimension but did marginally increase the likelihood of increasing the in Y‐dimension, though this did not lead to a significant improvement of the model overall. The null model showed that participants were more likely to not change in the X‐dimension and to increase in the Y‐dimension. A subset of the youngest children (15% of 3‐year‐olds) built structures identical to the experimenter, while all older children built something different from the experimenter. Compared with the baseline structure (a column four blocks tall), most participants (82%) tended to increase in the Y‐dimension only (e.g., a single column of five or more blocks high) but some (11%) increased in both dimensions (e.g., two adjacent columns each five blocks high) and this did not differ significantly by age.

### Supplementary results

The results presented here show that there is an Age effect for the negative polarity words (*smaller* and *shorter*). Our interpretation is that when children made their own structure with dimensional increases, that this was due to a misinterpretation of the word itself. However, an alternative possibility is that children were potentially ignoring the task instructions and instead engaging in a more general block‐building game with the experimenter (where the usual schema of block‐building is to build bigger things). We think this is unlikely, as trials were excluded if the participant was clearly ignoring task instructions (e.g., declaring that they would build *bigger*). To rule out this possibility though, we ran two supplementary analyses. First, we assumed that if children were ignoring the target words, there should be no difference between *smaller* and *shorter*, in children's likelihood of building structures that were actually bigger. However, if the children were misinterpreting the words, they may show a difference if they acquired the polarity aspect at different points in development. The results overall showed that children were more likely to build bigger structures with the word *shorte*r than the word *smaller*, suggesting that they were attending to the words. Next, we analyzed the confirmation questions asked at the end of each trial, in which the experimenter asked the child which of the two structures (the child's or experimenter's) matched the target word. If children understood the meaning of the word but were distracted by the block‐building, we would expect that children would identify the experimenter's structure as *smaller/shorter* at the confirmation question. However, children overwhelmingly confirmed that their own (larger) structure was *smaller/shorter*, indicating that they had misinterpreted the word. The details of these analyses are in the Supporting Information.

## DISCUSSION

This study investigated how children's understanding of five size comparison words (*bigger*, *smaller*, *shorter*, *longer*, and *taller*) changed from three through 8 years of age. Our data showed that overall accuracy increased with age and that the different words showed different trajectories, both in the overall accuracy and in the types of structures children built. For *bigger*, children across the age range were highly accurate and tended to increase their structure in the same dimension that was greater in the experimenter's structure, but generally applied the word to both height and width dimensions. For *longer*, the youngest children were less accurate, but accuracy quickly improved. When the baseline structure was long, children tended to increase their structure in that same dimension, a pattern that became stronger with age, but when the baseline was tall, children showed more mixed responses across the age range, with most increasing in the same dimension, but a substantial number increasing in the X‐dimension. For both *smaller* and *shorter*, accuracy improved with age, particularly between 3 and 5 years. Younger children showed mixed responses, with a substantial number of 3‐ and 4‐year‐olds increasing the structure in the same dimension that was greater in the baseline structure but with age, becoming more likely to decrease that dimension. For *taller*, accuracy improved with age. Younger children tended to increase their structures in the same dimension that was greater in the baseline structure. When the baseline structure was tall, children tended to increase in that same dimension, and this did not significantly differ with age. When the baseline was long, children initially increased in that same dimension, but became gradually more likely to increase in height with age.

Overall, these results show that children do learn the meaning of different comparative words on different trajectories. In particular, some previous work (Layton & Stick, 1979; Smith & Sera, 1992) showed that children, from quite early on, know the meanings of both positive and negative polarity words, while other work (e.g., Bishop & Bourne, 1985) suggested that negative polarity words are learned later. Our findings are consistent with the idea that children's initial understanding of the meaning of these comparative words is incomplete, with only some of the key features that define the word meaning present initially and that the other key features are acquired later (Clark, 1973, 2022). In all of the words, children reliably built structures that differed from the baseline structures in size, indicating that the children had encoded that the words referred to size changes. However, younger children also tended to treat these words as synonymous with *bigger*, increasing the size of the structures, even when asked to make them smaller or shorter. One alternative possibility we considered was that some children might have ignored the experimenter's instructions, and instead just engaged in a more general block‐building game (which typically involves making things bigger), but this is unlikely in light of the supplementary analyses that showed different rates of bigger structures for *smaller* and *shorter*, and overwhelming confirmation of their own (larger) structures as *smaller/shorter* when asked a confirmation question. This suggests that they gradually acquired the polarity distinction, where the meaning of the word captured whether the size change was an increase or a decrease. Finally, the dimension attribute was acquired last, with children initially treating *bigger* and *taller* as synonymous, tending to increase in the same dimension that was greater in the baseline structure. With *bigger*, we found no evidence that one dimension was biased over the other (i.e., children built structures that increased in the X‐dimension and the Y‐dimension, with no clear changes across age). This is in contrast to some previous work (Bausano & Jeffrey, 1975; Maratsos, 1973) showing that children between the ages of 3 and 5 years switch to encode *bigger* as more likely to refer to an increase in height. Our results revealed that children's interpretations of *bigger* did not change across 3‐ to 8‐year‐olds. Children gradually acquired the dimensional attribute of *taller*, specifically building structures with greater height, regardless of the baseline structure. These findings are consistent with previous work showing a protracted development of other relational words, with the meanings becoming more precise with age. Children between the ages of 3‐ and 5 years gradually learn the meaning of the words *middle* and *between*, with no 3‐year‐olds knowing either word, most 5‐year‐olds knowing the word *between* and less than half of 5‐year‐olds knowing the more precise *middle*, with most treating *middle* and *between* as synonymous (Simms & Gentner, 2019). Similarly, 3‐year‐olds initially treat the quantifiers *more* and *less* as synonymous, referring to increases in quantity (Donaldson & Balfour, 1968) and it is not until several years later that *less* is reliably encoded as a decrease (Palermo, 1973). Our findings are consistent, with a gradual refinement of the meanings after 3 years of age.

This study also clarifies some of the mixed results found previously with comparative words. While some work showed robust skills even in children as young as 3 years (e.g., Layton & Stick, 1979), other work showed a more protracted development in the acquisition of comparative words (e.g., Bishop & Bourne, 1985). Importantly, however, these studies tended to not break down their analyses by individual words and tended to use complex syntax that might have also influenced children's performance beyond the words themselves. Here, we tested a wide age range of children, analyzed the data at the individual word level to determine if different words showed different developmental patterns, and simplified the verbal instructions to a short question with the target word at the end, to avoid the influence of complex syntax. The design also allowed for substantial flexibility in children's responses to demonstrate their individual interpretation of the word, by allowing children to build their own structures in response to the prompt. Future research can investigate whether different baseline structures change the interpretations of the words (e.g., do dimensional biases emerge when the baseline structure is matched on dimensions, as in a 2 × 2 structure? Do variations in the number of blocks in the baseline structure influence the structures children build?). This would allow for more detailed analyses of which factors influence the interpretations of the word meanings.

These findings also raise questions about how children's comparative skills develop. The acquisition of relational language has been proposed to support the acquisition of those relational concepts (e.g., Gentner et al., 2003, 2016), and indeed, previous work (Simms & Gentner, 2019) showed that children who had a better understanding of the words *middle* and *between* were better able to learn that a treasure was always hidden in the middle of two flags. Critically, however, the developmental trajectories of the acquisition of these comparative terms, in the current study at least, does not appear to reflect the frequencies of these structures in input to children. Specifically, a search of the UK corpora in the CHILDES database of conversations between adults and children revealed that the most frequent of our comparative words in maternal input to children was *bigger* (652), in line with our results. However, if acquisition were related only to input frequency, we would expect *smaller* (218) to be acquired earlier than *longer* (166). While we remain cautious as to the extent to which these corpus statistics reflect the linguistic input of the children in our study, in line with previous work, this suggests that the direction of causality of the relationship between conceptual and lexical acquisition is unclear. Specifically, does having a relational concept make it easier to learn that concept's label, or does learning the label drive the acquisition of the concept? Work is currently ongoing to investigate how comparative words link to the acquisition of comparative concepts. The diverse trajectories that we see in comparative words can also be foundational in developing computational models for understanding language and relational development (Doumas et al., 2008, 2022; Twomey & Westermann, 2017).

Importantly, our sample consisted of English‐learning children who participated in a public museum in a large city in England. While we do present the results including multilingual children in the Supporting Information, our study was not designed to test for differences due to language background, and more directed research is needed to examine how the language background shapes the acquisition of abstract words. Further research is also needed to investigate the development of comparative language skills in children learning other languages, particularly languages that highlight different aspects of size comparison, which can help clarify how language integrates with concepts over development (e.g., Hespos & Spelke, 2004). In addition, the use of relational language has been linked to other skills including analogical reasoning (Frausel et al., 2020; Silvey et al., 2017) and mathematics (Richland et al., 2017; Rittle‐Johnson & Star, 2007, 2009) and future research can investigate the role that comparative language plays in the development of these skills.

In summary, in the first fine‐grained study of the trajectory of acquisition of five different comparative words, we show that children's interpretation of the meaning of size comparison words changes across development. Children initially learn that these words refer to size comparisons generally, then distinguish between positive and negative types of size comparisons, and then attend to the specific dimension involved in these changes. Understanding how children acquire these words can shed light on how children's relational thinking and language interact and how these skills influence other areas of development (e.g., spatial and mathematical skills).

## AUTHOR CONTRIBUTIONS

Alissa L. Ferry: conceptualization; methodology; investigation; formal analysis; data curation; visualization; writing—original draft preparation; writing—review and editing; supervision. Mia Corcoran: conceptualization; methodology; investigation. Emily Williams: conceptualization; methodology; investigation. Sheila M. Curtis: investigation. Cathryn J. Gale: investigation. Katherine E. Twomey: conceptualization; methodology; investigation; writing—review & editing; supervision.

## Supporting information


Data S1.

## Data Availability

The materials, data and analytic code necessary to reproduce the analyses presented here are publicly accessible at https://osf.io/ekvqc/. The analyses presented here were not preregistered.
