# Supplementary material for: Bigger versus smaller: Children's understanding of size comparison words becomes more precise with age
Source: Child Dev. 2024 Nov 1;96(2):492–507. doi: 10.1111/cdev.14182 (PMC11868677; doi:10.1111/cdev.14182)
Supplement: Supplementary file 1 — Data S1. [file CDEV-96-492-s001.docx]

Supplementary Information

**Supplementary Figure 1**

*The Histogram of the Age of the Included Participants in the Main Analysis*


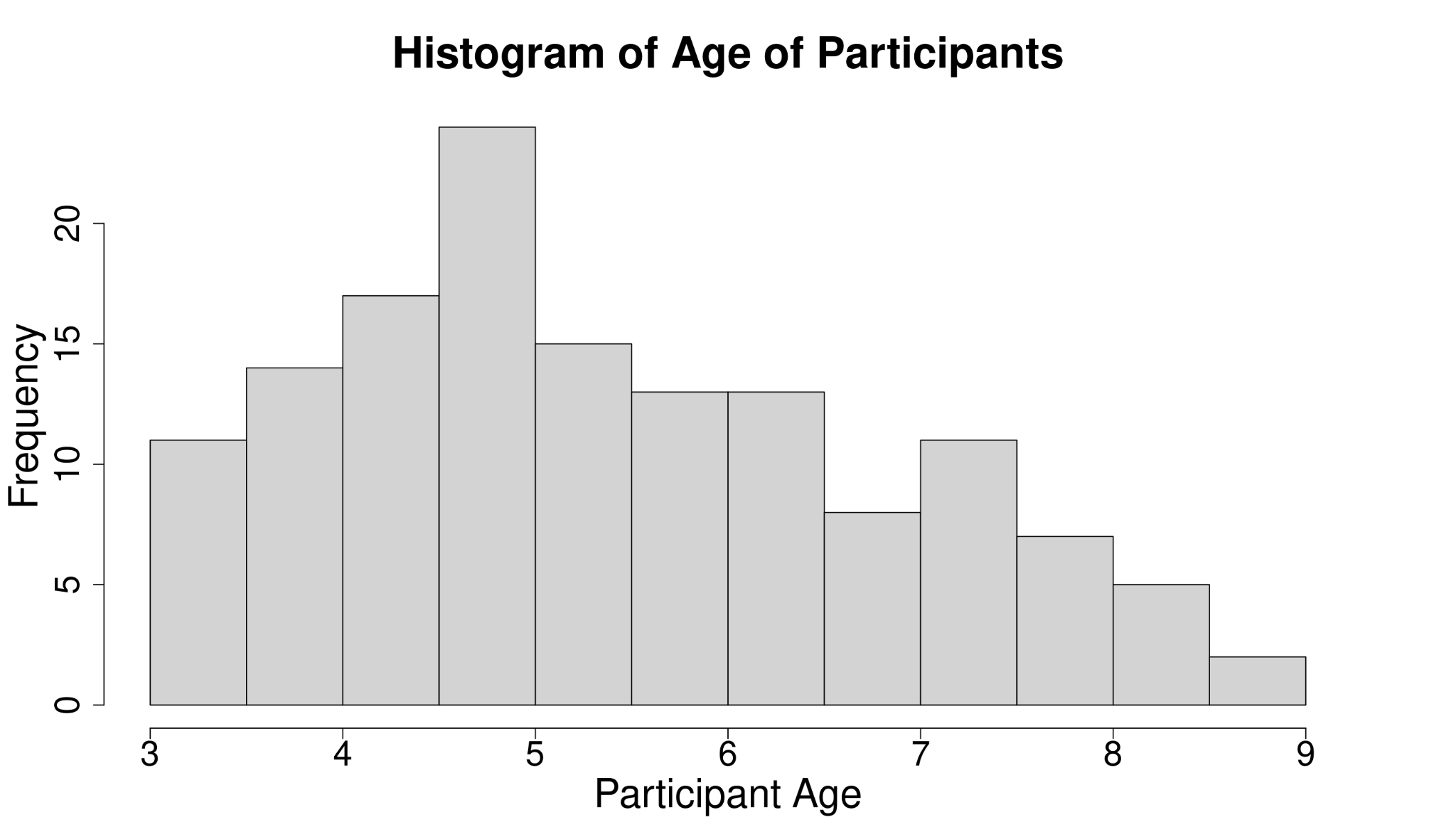


*Note:* The age bins are six-month-intervals.

**Supplementary Methods: An example interpretation of the Condition Structure Analysis**

The ordinal models model the odds-ratios and different cut-points. This is relatively straightforward when there are two cut points. For example, if the experimenter’s structure was Long (4 blocks in the X-dimension and 1 in the Y-dimension), there are only two options for the Y-dimension: the participant can use the same number of blocks (only one block tall) or they can use more. This is represented as the threshold of nochangeY|increaseY. If children tend to not change in the Y-dimension much more than than they increased in the Y-dimension, you get an odds-ratio of greater than one (e.g., if 75% of the time there was no change and 25% of the time there was an increase, you get an odds ratio of 3; the no change was three times as likely). Similarly, if participants were more likely to increase their structure rather than not change it, you would get an odds-ratio of less than one (e.g., if 25% of the time there was no change and 75% of the time there was an increase, you get an odds ratio of .33; the no change was one-third times as likely). If participants were equally likely to not change in the Y-dimension and increase in the Y-dimension, you would get an odds ratio of around 1 (e.g., if 50% of the time there was no change and 5o% of the time there was an increase, you get an odds ratio of 1).

The interpretation is slightly more complex when there are more ordinal options, as the model provides odds-ratios for each cut-point (and not for individual outcomes). For example, if the experimenter’s structure was Long (4 blocks in the X-dimension and 1 in the Y-dimension), there are three options for the X-dimension: the participant can use fewer blocks in the X-dimension, the same number of blocks, or they can use more. The model provides the odds-ratios for the two cut-off points, comparing the odds of falling on either side of that cutoff point. The decreaseX|nochangeX odds-ratio provides the ratio for fewer blocks compared to either no change or an increase in blocks (framing it as a ratio of decreaseX|(nochangeX or increaseX) may ease the interpretation) The nochangeX|increaseX odds-ratio provides the ratio for fewer blocks or no change in blocks compared to an increase in blocks (framing it as a ration of (decreaseX or nochangeX)|increaseX may ease the interpretation). As an example, if participants generally increased a structure in the X-dimension from a Long baseline we would expect the decreaseX|nochangeX OR to be below 1 (because there are very few trials with a decrease in X, but many more that are either no change or increase) and the nochangeX|increaseX to also be below 1 (because there are very few trials that are decrease or no change but many that are an increase). If participants generally did not change a structure in the X-dimension from a Long baseline we would expect the decreaseX|nochangeX OR to be below 1 (because there are many trials with a decrease in X, but few that are either no change or increase) and the nochangeX|increaseX to also be above 1 (because there are many trials that are decrease or no change but few that are an increase). Similarly, if participants generally decreased a structure in the X-dimension from a Long baseline we would expect the decreaseX|nochangeX OR to be above 1 (because there are many trials with a decrease in X, but few that are either no change or increase) and the nochangeX|increaseX to also be above 1 (because there are many trials that are decrease or no change but few that are an increase).

As a concrete example, the Long-Bigger Base model shows that the decreaseX|nochangeX coefficient is less than 1, indicating that decreaseX is less likely than the combined nochangeX and increaseX. The nochangeX|increaseX coefficient is also less than 1, indicating that decreaseX and nochangeX are combined less likely than increaseX. Thus, most trials include an increase in the X-dimension. In the Y-dimension, nochangeY|increaseY is greater than 1, indicating that nochangeY is more frequent than increaseY. Thus, in Long-Bigger, participants generally tend to increase in the X-dimension and not change in the Y-dimension.

**Supplementary Analyses**

The main analyses showed that some participants built larger structures when asked to build ‘smaller’ or ‘shorter’ ones. We suggest that this is due to children initially having a different meaning linked to the word (e.g., that ‘smaller’ means more, rather than less). However, an alternative explanation is that these children might be ignoring the game instructions (“Can you build me one that is smaller?”) and instead just engaging in a block building schema, that when you build things with blocks, the goal is often to build things bigger. To rule out this hypothesis we did two additional analyses

More Blocks Analysis

First, we investigated whether there were differences between ‘smaller’ and ‘shorter’ in the rate of building structures with more than four blocks (the number in the baseline structure). If participants were distracted by the task and ignoring the word requested, the rate of building structures should not differ between words. However, if there were differences between the words, that suggests that the participants are attending to the task and the words and that the participants are building larger structures based on their interpretation of the word. We included Age in the model as we also hypothesized that children would be less likely to build with more than four blocks with increasing age.

Here, we looked specifically at the negative polarity words and coded whether children used more than four blocks in their own structure. The analysis was conducted using binomial generalized linear mixed effects models in the *lme4* package in R [(Bates et al., 2015)](https://www.zotero.org/google-docs/?YGcxng). The outcome variable was whether children used more blocks than in the baseline and random intercepts were set for each participant as participants completed multiple trials. Three models were compared: a baseline model with no fixed effects, a model with Age (as a continuous variable, centered so that 0 represented the youngest point in our time window, 3-years-old, to ease interpretation) and Word (as a categorical variable, with *shorter* as the reference category) as fixed effects, and a model with their interaction. The best model was identified by the likelihood ratio test for model comparisons [(Pinheiro & Bates, 2000)](https://www.zotero.org/google-docs/?LEvtcP). We used the R package *lmerTest* [(Kuznetsova et al., 2017)](https://www.zotero.org/google-docs/?r18kyd) to calculate *p*-values. Raw model estimates are on the log-odds scale and are presented here as odds ratios (ORs) to ease interpretation. ORs greater than 1 indicate a greater likelihood of a correct response compared to an incorrect response, less than 1 indicating a greater likelihood of an incorrect response, and ORs around 1 indicating an equal likelihood. Age coefficients less than 1 indicate that the number of structures built with more than four blocks decreases with age. Posthoc tests of the estimated marginal means of the intercepts and age effects for each word were done using the *emmeans* R package [(Lenth et al., 2019)](https://www.zotero.org/google-docs/?RzKvGY), with Holm-Bonferroni adjustments.

The data showed that of 263 *smaller* trials, 28 (10.6%) of the participant structures included more than four blocks and that of 259 *shorter*, 46 (17.8%) of the participant structures included more than four blocks. The model with main effects of Age and Word was a better fit than the baseline model (*p* < .0001) and the model with the interaction of Age and Word did not further improve the model (*p* = .60) The models are available in Supplementary Table 1. The predicted accuracy for the Age and Word model is in Supplementary Figure 2. Because there was an effect of Age and Word, we compared the estimated marginal means at the start of the age window (three-year-olds) to assess the initial accuracy for each word and the slopes to assess how the accuracy changed with age. Three-year-old children were equally likely to build structures with more than four blocks as they were to build structures with four or fewer blocks for both shorter (OR = 1.74, *p* = .55) and smaller (OR = .656, *p* = .55). However, there was a significant difference between them, with *shorter* resulting in more structures with more than four blocks than *smaller* (*p* < .005). The likelihood of building the larger structures overall decreased with Age (OR = .236, *p* < .0001).

In sum, three-year-olds are more likely to build bigger structures when the word was *shorter* than when it was *smaller* and this decreased with age, at the same rate for both words. Thus suggests that tendency to build things bigger with negative polarity words was not due to a more general tendency to build things bigger, but is instead related to their understanding of the word.

**Supplementary Table 1**

*Summary of the Binomial Generalized Linear Mixed-Effects Models for Building Larger Structures.*

|  | **Null Model (NullModel_Blocks)** | | | **Age and Word Main Effects (Age_Word_Model_Blocks)** | | | **Age and Word Interaction (Age_WordModel_Blocks)** | | |
| --- | --- | --- | --- | --- | --- | --- | --- | --- | --- |
| *Predictors* | *Odds Ratios* | *CI* | *p* | *Odds Ratios* | *CI* | *p* | *Odds Ratios* | *CI* | *p* |
| (Intercept) | 0.02 | 0.00 – 0.15 | **<0.001** | 1.74 | 0.64 – 4.71 | 0.276 | 1.96 | 0.65 – 5.92 | 0.230 |
| Age centered |  |  |  | 0.24 | 0.14 – 0.40 | **<0.001** | 0.22 | 0.12 – 0.41 | **<0.001** |
| Word [smaller] |  |  |  | 0.38 | 0.19 – 0.74 | **0.005** | 0.28 | 0.08 – 1.02 | 0.053 |
| Age centered X Word [smaller] |  |  |  |  |  |  | 1.23 | 0.58 – 2.61 | 0.593 |
| **Random Effects** | | | | | | | | | |
| σ^2^ | 3.29 | | | 3.29 | | | 3.29 | | |
| τ_00_ | 11.54 _ParticipantID_ | | | 3.05 _ParticipantID_ | | | 3.11 _ParticipantID_ | | |
| ICC | 0.78 | | | 0.48 | | | 0.49 | | |
| N | 139 _ParticipantID_ | | | 139 _ParticipantID_ | | | 139 _ParticipantID_ | | |
| Observations | 522 | | | 522 | | | 522 | | |
| Marginal R^2^ / Conditional R^2^ | 0.000 / 0.778 | | | 0.413 / 0.695 | | | 0.400 / 0.692 | | |
| AIC | 373.270 | | | 324.836 | | | 326.554 | | |

**Supplementary Figure 2**

*Summary of the Binomial Generalized Linear Mixed-Effects Models for Building Larger Structures.*

*
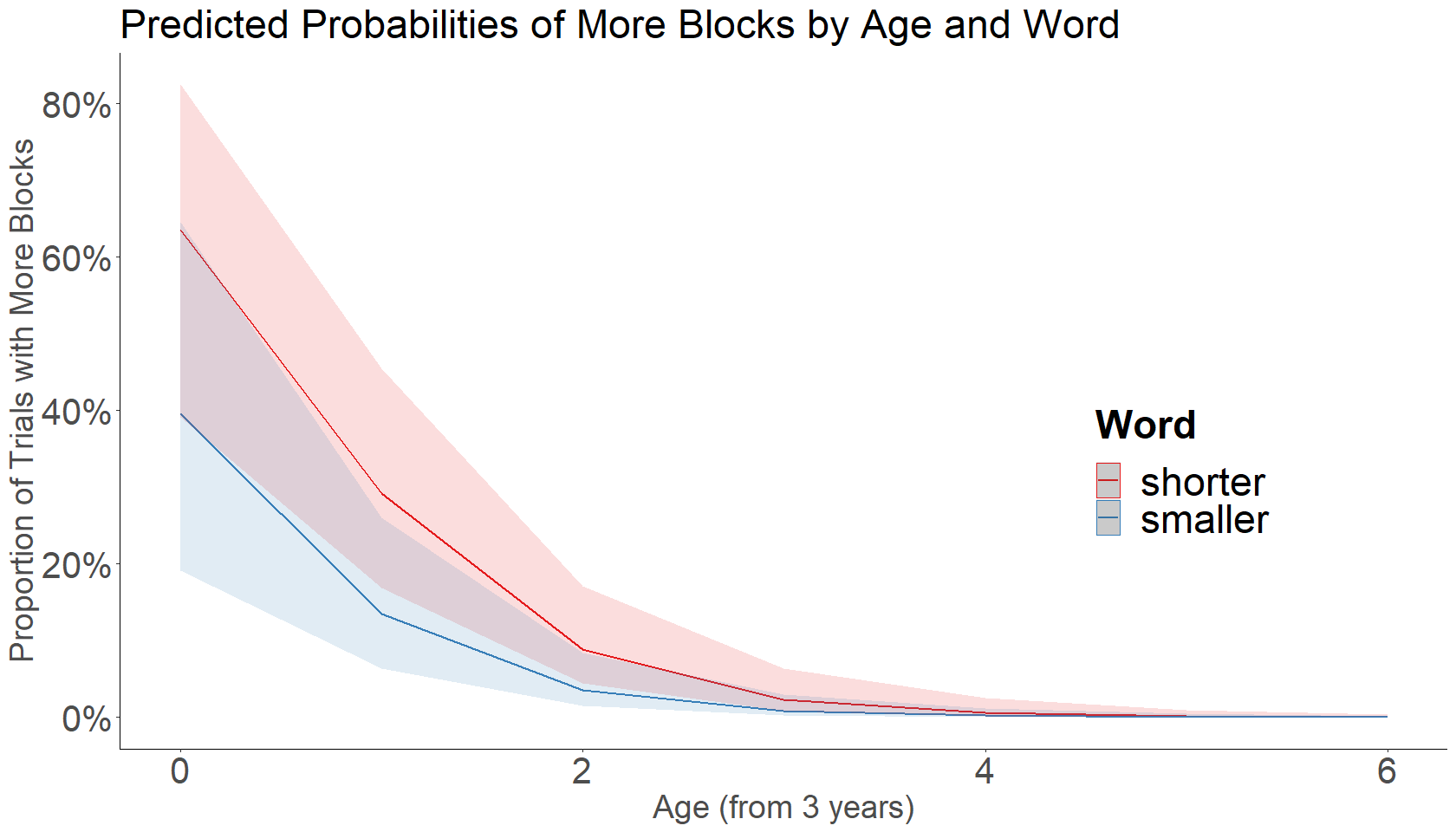
*

*Note:* The model predicted probabilities of building structure with more than four blocks, for each word, across the age range.

Certainty Forced Choice Analysis

Second, we investigated whether children were confident in their structures. After children build their structure, the experimenter asked them a confirmation question (‘Which one is [target word]?’) to which the child typically indicated one of the structures (e.g., pointing to their own, saying ‘Mine’). Children were coded as certain in their response if they selected their own structure and uncertain if they selected the experimenter’s or said that the structures were the same. If children were distracted by the building task, but knew the meaning of the words, we would expect that children would correct themselves at the confirmation question (i.e., that when asked, ‘Which one is smaller?’, they should correctly identify the experimenter’s as smaller, if their own structure had more blocks). If children were misinterpreting the words and thought their structures were correct, they should confirm their response. We examined how often children confirmed their response, if that differed based on whether they were correct initially, and whether Age changed their responses.

We looked specifically at *smaller* and  *shorter* trials where children faced a forced-choice paradigm in the confirmation question (i.e., their structure was different from the experimenter’s) and they provided a response (e.g., if a child knocked their structure down before the question could be asked, the trial was excluded). This included a total of 470 trials. Here we were interested in whether the certainty in their responses differed depending on whether children were correct or incorrect based on the structure they built. In (434 trials (92.3%), children indicated that their own structure matched the word. Given that there were a small number of trials in which children expressed uncertainty (36 trials), we opted to use a generalized linear model with the Firth correction method to reduce the small-sample bias in the maximum likelihood estimation [(Kosmidis, 2023)](https://www.zotero.org/google-docs/?fgQ0Lq).

The outcome variable was certainty. Three models were compared: a baseline model with no fixed effects, a model with Age (as a continuous variable, centred so that 0 represented the youngest point in our time window, 3-years-old, to ease interpretation) and Correct (as a categorical variable, with Correct as the reference category) as fixed effects, and a model with their interaction. The best model was identified by the likelihood ratio test for model comparisons [(Pinheiro & Bates, 2000)](https://www.zotero.org/google-docs/?BxYTzu). We used the R package *lmerTest* [(Kuznetsova et al., 2017)](https://www.zotero.org/google-docs/?N5jE5j) to calculate *p*-values. Raw model estimates are on the log-odds scale and are presented here as odds ratios (ORs) to ease interpretation. Posthoc tests of the estimated marginal means of the intercepts and age effects for each word were done using the *emmeans* R package [(Lenth et al., 2019)](https://www.zotero.org/google-docs/?ZpFo7S), with Holm-Bonferroni adjustments.

The model with main effects of Age and Correct was a better fit than the baseline model (*p* < .0005) and the model with the interaction of Age and Correct did further improve the model (*p* < .05) The models are available in Supplementary Table 2. The predicted accuracy for the Age and Correct interaction model is in Supplementary Figure 3. Because there was an interaction of Age and Correct, we compared the estimated marginal means at the start of the age window (three-year-olds) to assess the initial accuracy for each word and the slopes to assess how the accuracy changed with age. Three-year-olds showed no difference in certainty (*p* = .70) when they were both correct (OR = 3.00) and incorrect (OR = 2.32) but there was an Age and Correct interaction (*p* < .05), with children becoming more certain when they were correct with age (OR = 2.61, *p* < .001) but showing no change in certainty when they were incorrect (OR = 1.00, *p* = .98)

In sum, children were overwhelmingly certain in their responses both when they were correct and when they were incorrect (e.g., confirming that their structure made with more than four blocks was *smaller* than the experimenter’s). While there was increasing certainty with age when children were correct, but not when they were incorrect, children in general, confirmed their responses when asked in a forced choice paradigm. This again suggests that tendency to build things bigger with negative polarity words was not due to a more general tendency to build things bigger, but is instead related to their understanding of the word.

**Supplementary Table 2**

*Summary of the Generalized Linear Models for the Confirmation Question Certainty*

|  | **Null Model Certainty** | | | **Age and Word Main Effects** | | | **Age and Word Interaction** | | |
| --- | --- | --- | --- | --- | --- | --- | --- | --- | --- |
| *Predictors* | *Odds Ratios* | *CI* | *p* | *Odds Ratios* | *CI* | *p* | *Odds Ratios* | *CI* | *p* |
| (Intercept) | 23.42 | 14.30 – 38.36 | **<0.001** | 5.61 | 2.35 – 13.38 | **<0.001** | 3.00 | 1.16 – 7.75 | **0.023** |
| Correct [Incorrect] | 0.10 | 0.05 – 0.21 | **<0.001** | 0.20 | 0.09 – 0.44 | **<0.001** | 0.77 | 0.21 – 2.83 | 0.698 |
| Age centered |  |  |  | 1.83 | 1.26 – 2.65 | **0.001** | 2.61 | 1.57 – 4.34 | **<0.001** |
| Correct [Incorrect] X Age centered |  |  |  |  |  |  | 0.38 | 0.18 – 0.81 | **0.011** |
| Observations | 470 | | | 470 | | | 470 | | |
| R^2^ Tjur | 0.114 | | | 0.126 | | | 0.138 | | |

**Supplementary Figure 3**

*Summary of the Binomial Generalized Linear Models for Confirmation Question Certainty.*

*
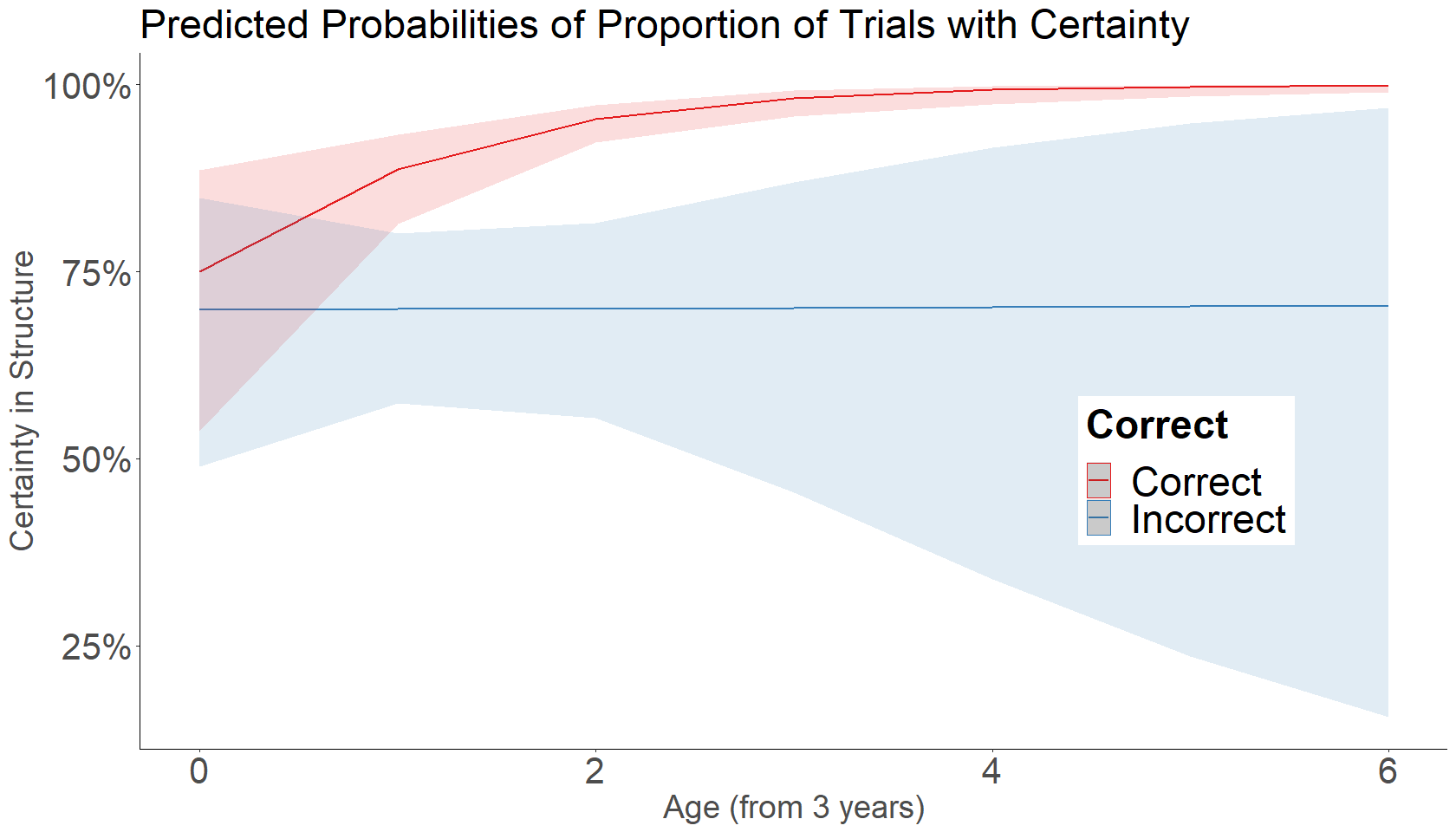
*

**Analysis of the Dataset including Multilingual Participants**

The main analysis looked specifically at children who were monolingual English speakers. Because we were uncertain of whether language exposure would influence the task performance, we opted to exclude multilingual participants. Here, we provide an analysis of the entire dataset. The goal of the study was not to compare monolingual and multilingual children and the numbers in each group are not balanced, nor did we control for bilingual characteristics (e.g., specific languages, proportion of English). Thus we do not compare the groups. Rather, we analyse the full data set, which more accurately represents the diverse population of children learning English. Demographics data showed that 19 different languages besides English were spoken among participants, with Spanish, Polish, Arabic, and Punjabi the most common.

Here, 168 children were included in the final analysis and 36 were excluded for: refusing to participate after the caregiver signed the paperwork (5), experimenter error (1), missing demographics information (5), the presence of hearing/visual impairments or a developmental delay (19), or not understanding the task instructions in English (6). The remaining participants were an average of 5.5 years (range: 3 years, 0 months to 8 years, 11 months, see Supplementary Figure 4) and included 79 boys (47%) and 89 girls (53%). Data and data processing information for the multilingual dataset are available at the same OSF Repository.

**Supplementary Figure 4**

*The Histogram of the Age* *of the Included Participants in the Multilingual Analysis*


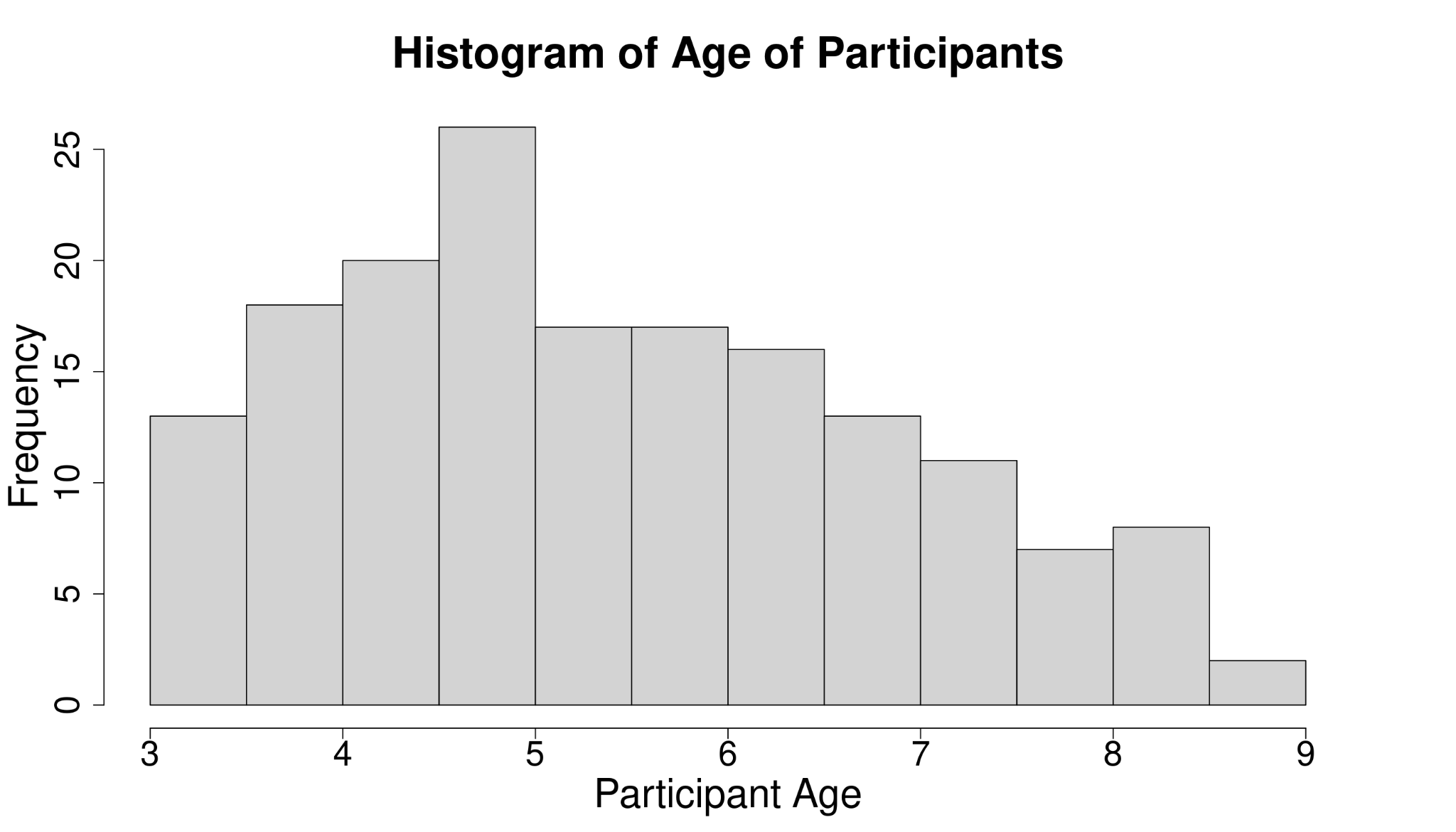


*Note:* The age bins are six-month-intervals.

The same analytical procedure was used as in the main analysis. The Accuracy results are presented in Supplementary Figure 5 and Supplementary Table 3. The Structure results are presented in Supplementary Figure 6 and Supplementary Table 4. The results are largely consistent with the monolingual dataset. The notable differences are: 1) in the Accuracy analysis, the initial intercept for *longer* is higher, though the overall model results maintain the same global patterns; 2) In the Structure analysis, the Age effect in the Tall-Taller condition was not significant in the main analysis (*p* = .01, with a multiple comparison adjusted threshold of .005) and is significant with the dataset included here.

**Supplementary Figure 5**

*The Accuracy for Each Word Across the Age Range of the Included Participants in the Multilingual Analysis*


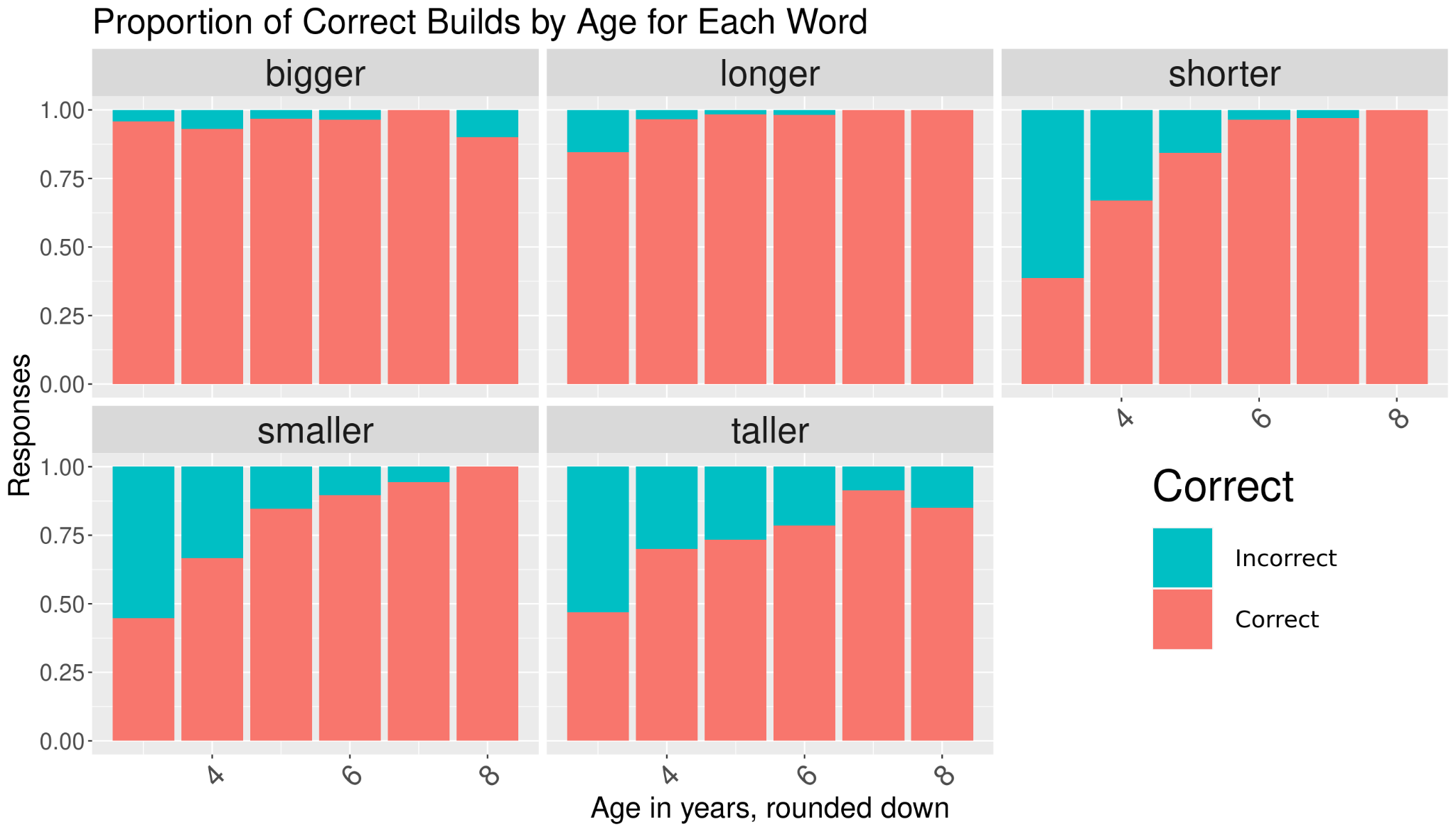


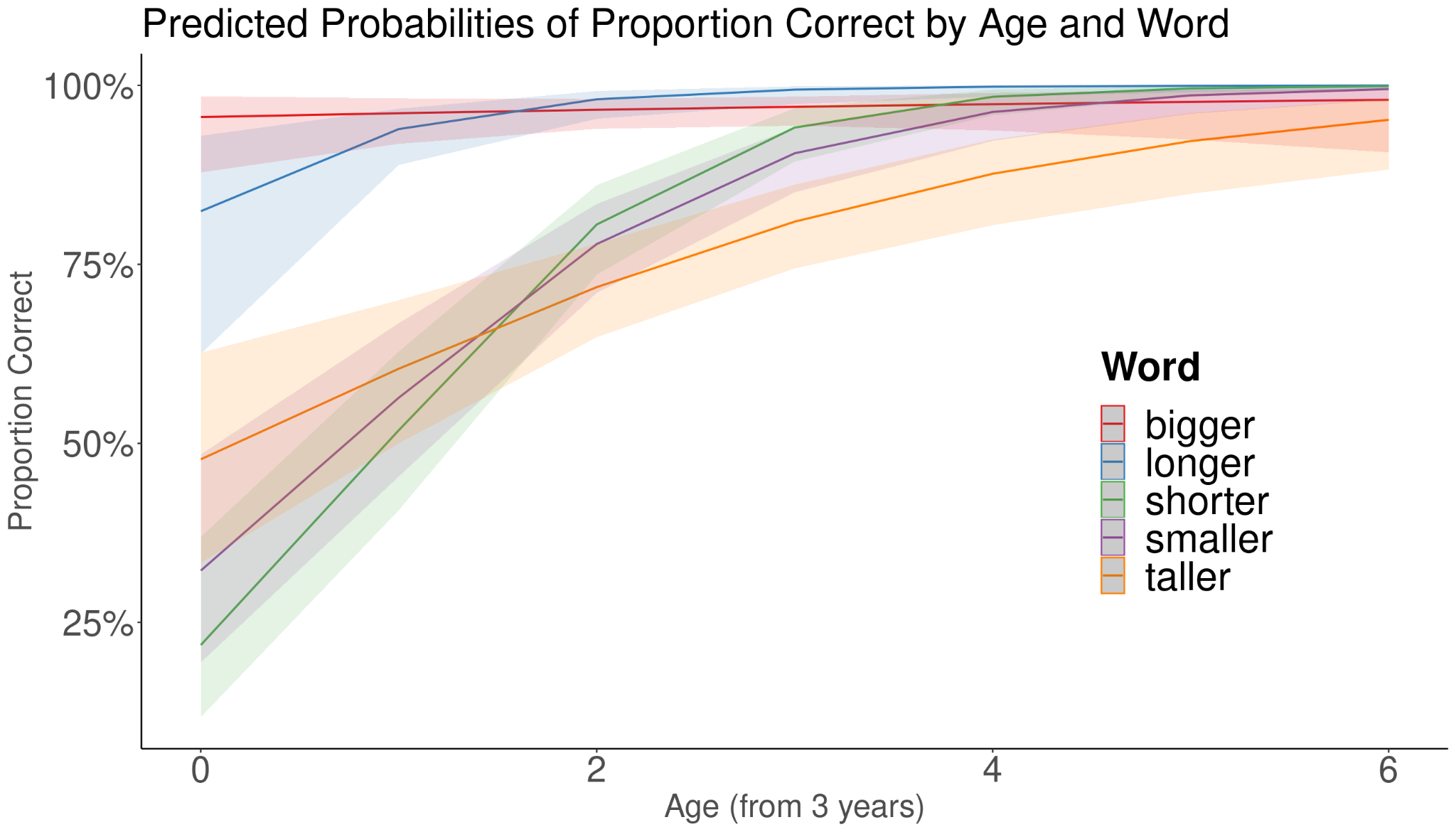


*Note:* Top) The proportions of correct and incorrect trials for each word , binned by age (rounded down). Bottom) The model predicted probabilities of building accuracy for each word, across the age range.

**Supplementary Table 3**

*Summary of the Binomial Generalized Linear Mixed-Effects Models for the trial accuracy.*

|  | **Null Model (NullModel_Accuracy)** | | | **Age and Word Main Effects (Age_Word_Model_Accuracy)** | | | **Age and Word Interaction (Age_WordModel_Accuracy)** | | |
| --- | --- | --- | --- | --- | --- | --- | --- | --- | --- |
| *Predictors* | *Odds Ratios* | *CI* | *p* | *Odds Ratios* | *CI* | *p* | *Odds Ratios* | *CI* | *p* |
| (Intercept) | 7.15 | 5.50 – 9.30 | **<0.001** | 6.36 | 3.26 – 12.42 | **<0.001** | 21.58 | 7.22 – 64.50 | **<0.001** |
| Word [longer] |  |  |  | 1.17 | 0.52 – 2.62 | 0.706 | 0.22 | 0.05 – 0.91 | **0.037** |
| Word [shorter] |  |  |  | 0.11 | 0.06 – 0.22 | **<0.001** | 0.01 | 0.00 – 0.05 | **<0.001** |
| Word [smaller] |  |  |  | 0.11 | 0.06 – 0.21 | **<0.001** | 0.02 | 0.01 – 0.08 | **<0.001** |
| Word [taller] |  |  |  | 0.08 | 0.04 – 0.15 | **<0.001** | 0.04 | 0.01 – 0.14 | **<0.001** |
| Age centered |  |  |  | 2.21 | 1.85 – 2.64 | **<0.001** | 1.14 | 0.76 – 1.71 | 0.511 |
| Word [longer] X Age centered |  |  |  |  |  |  | 2.87 | 1.28 – 6.43 | **0.011** |
| Word [shorter] X Age centered |  |  |  |  |  |  | 3.37 | 1.97 – 5.75 | **<0.001** |
| Word [smaller] X Age centered |  |  |  |  |  |  | 2.37 | 1.46 – 3.87 | **0.001** |
| Word [taller] X Age centered |  |  |  |  |  |  | 1.46 | 0.94 – 2.27 | 0.095 |
| **Random Effects** | | | | | | | | | |
| σ^2^ | 3.29 | | | 3.29 | | | 3.29 | | |
| τ_00_ | 1.38 _ParticipantID_ | | | 0.81 _ParticipantID_ | | | 0.89 _ParticipantID_ | | |
| ICC | 0.30 | | | 0.20 | | | 0.21 | | |
| N | 168 _ParticipantID_ | | | 168 _ParticipantID_ | | | 168 _ParticipantID_ | | |
| Observations | 1561 | | | 1561 | | | 1561 | | |
| Marginal R^2^ / Conditional R^2^ | 0.000 / 0.295 | | | 0.394 / 0.514 | | | 0.441 / 0.560 | | |
| AIC | 1323.833 | | | 1097.548 | | | 1075.345 | | |

**Supplementary Figure 6**

*Heat maps of each of the ten conditions.*

*
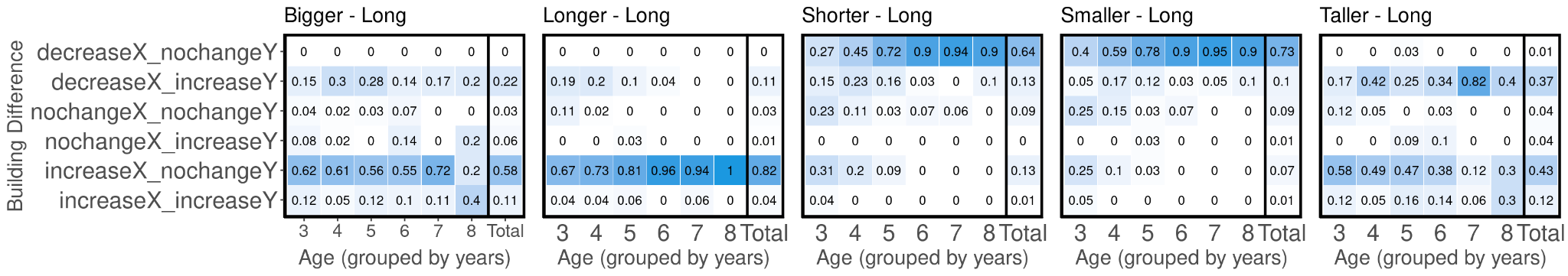
*
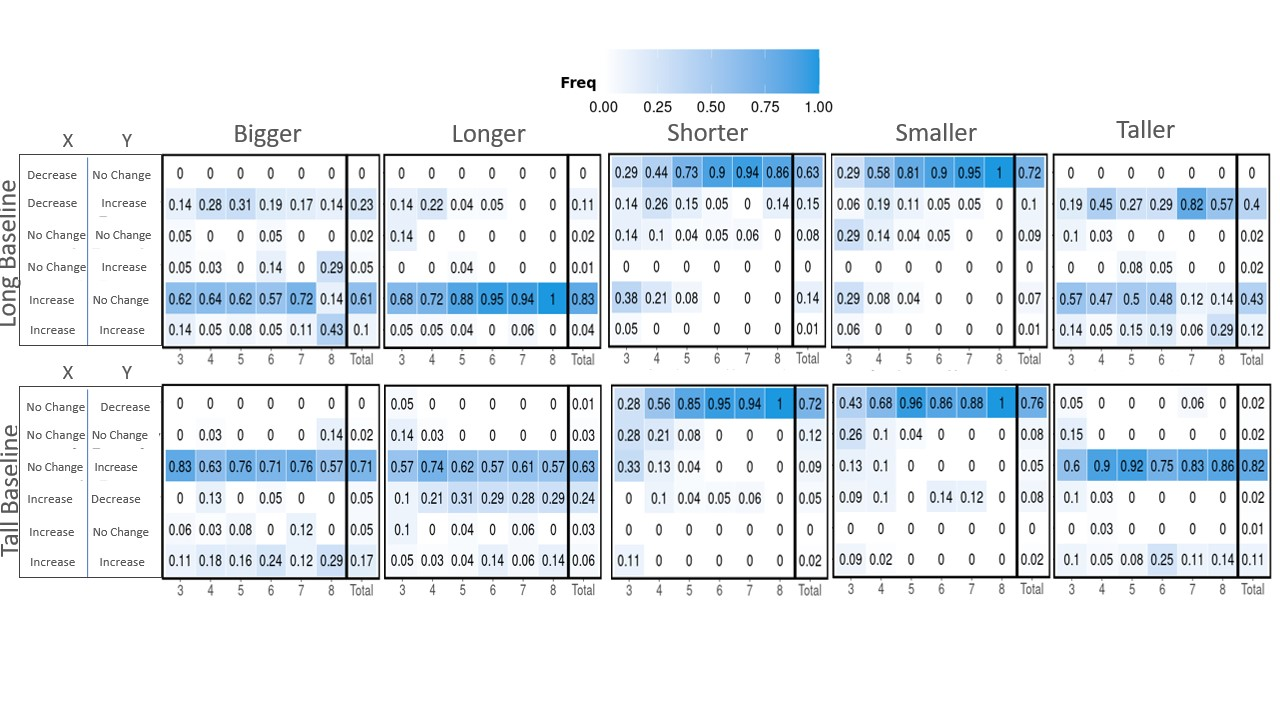


*
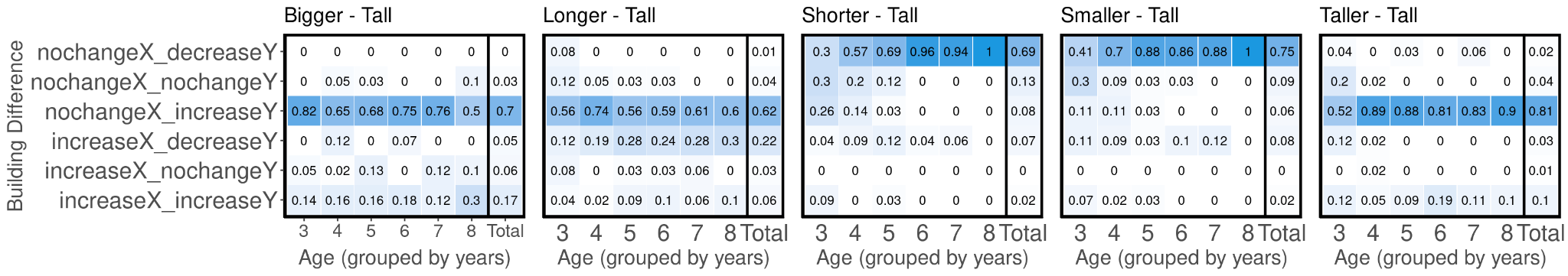
*

*Note*: Each plot shows the proportion of the type of structure for each age bin (age in years, rounded down). Baseline long structures are plotted on the top and baseline tall structures are plotted on the bottom.

**Supplementary Table 4**

*Summary of the Multivariate Ordinal Regression Models for structures built in each condition.*

| Model Comparisons for Each Word in the "Long" Condition | | | | | | | | | | |
| --- | --- | --- | --- | --- | --- | --- | --- | --- | --- | --- |
|  | Bigger | | Longer | | Shorter | | Smaller | | Taller | |
|  | Null Model | Age Model | Null Model | Age Model | Null Model | Age Model | Null Model | Age Model | Null Model | Age Model |
| X decreaseX\|nochangeX | 0.464**** | 0.491*** | 0.273**** | 0.661 | 2.575**** | 0.691 | 2.114**** | 0.671 | 0.749** | 0.478*** |
|  | [0.371, 0.581] | [0.325, 0.741] | [0.191, 0.390] | [0.335, 1.301] | [2.027, 3.271] | [0.399, 1.196] | [1.697, 2.635] | [0.417, 1.080] | [0.609, 0.921] | [0.313, 0.728] |
| X nochangeX\|increaseX | 0.615**** | 0.656* | 0.343**** | 0.799 | 4.284**** | 1.323 | 3.046**** | 1.06 | 0.917 | 0.588* |
|  | [0.502, 0.754] | [0.437, 0.986] | [0.270, 0.435] | [0.456, 1.400] | [3.162, 5.806] | [0.770, 2.271] | [2.369, 3.916] | [0.682, 1.650] | [0.753, 1.117] | [0.392, 0.881] |
| Y nochangeY\|increaseY | 1.312** | 1.581* | 2.803**** | 1.498 | 3.363**** | 2.594*** | 2.956**** | 1.77* | 0.921 | 1.861** |
|  | [1.071, 1.608] | [1.053, 2.373] | [2.090, 3.758] | [0.852, 2.635] | [2.574, 4.394] | [1.474, 4.565] | [2.309, 3.784] | [1.136, 2.759] | [0.751, 1.129] | [1.202, 2.883] |
| corr X Y | -0.925**** | -0.925**** | -0.925**** | -0.925**** | -0.122 | -0.186 | -0.444** | -0.647**** | -0.916**** | -0.917**** |
|  | [-1.003, -0.847] | [-1.003, -0.847] | [-1.078, -0.772] | [-1.115, -0.735] | [-0.511, 0.268] | [-0.589, 0.217] | [-0.758, -0.129] | [-0.914, -0.381] | [-0.990, -0.841] | [-0.991, -0.844] |
| Age(X) |  | 1.027 |  | 1.462* |  | 0.528**** |  | 0.574**** |  | 0.838* |
|  |  | [0.887, 1.188] |  | [1.088, 1.964] |  | [0.407, 0.684] |  | [0.458, 0.720] |  | [0.730, 0.962] |
| Age(Y) |  | 1.073 |  | 0.784* |  | 0.9 |  | 0.794** |  | 1.333*** |
|  |  | [0.932, 1.236] |  | [0.619, 0.993] |  | [0.732, 1.107] |  | [0.667, 0.944] |  | [1.142, 1.555] |
| **Likelihood Ratio Test** |  | **n.s.** |  | **p < .0001** |  | **p < .00001** |  | **p < .00001** |  | **p < .001** |
| Num.Obs. | 159 | 159 | 158 | 158 | 151 | 151 | 158 | 158 | 155 | 155 |
| AIC | 383.18 | 383.17 | 225.91 | 211.3 | 288.27 | 257.71 | 348.65 | 304.39 | 404.98 | 393.52 |
| BIC | 395.77 | 402.31 | 238.48 | 230.4 | 300.67 | 276.56 | 361.22 | 323.49 | 417.47 | 412.51 |
| * p < 0.05, ** p < 0.01, *** p < 0.001, **** p < .0001 | | | | | | | | | | |

| Model Comparisions for Each Word in the "Tall" Condition | | | | | | | | | | |
| --- | --- | --- | --- | --- | --- | --- | --- | --- | --- | --- |
|  | Bigger | | Longer | | Shorter | | Smaller | | Taller | |
|  | Null Model | Age Model | Null Model | Age Model | Null Model | Age Model | Null Model | Age Model | Null Model | Age Model |
| X nochangeX\|increaseX | 1.832**** | 2.155** | 1.587**** | 2.237*** | 3.503**** | 2.639*** | 3.801**** | 2.816*** | 3.016**** | 2.778**** |
|  | [1.473, 2.278] | [1.342, 3.463] | [1.289, 1.954] | [1.447, 3.460] | [2.681, 4.578] | [1.580, 4.407] | [2.859, 5.053] | [1.533, 5.172] | [2.342, 3.883] | [1.682, 4.588] |
| Y decreaseY\|nochangeY | 0.185**** | 0.182**** | 0.492**** | 0.456*** | 2.621**** | 0.862 | 2.066**** | 0.451** | 0.184**** | 0.394* |
|  | [0.129, 0.266] | [0.107, 0.309] | [0.395, 0.613] | [0.299, 0.696] | [2.079, 3.304] | [0.519, 1.430] | [1.656, 2.577] | [0.255, 0.800] | [0.129, 0.263] | [0.182, 0.853] |
| Y nochangeY\|increaseY | 0.331**** | 0.325**** | 0.622**** | 0.585** | 4.061**** | 1.467 | 3.523**** | 0.93 | 0.263**** | 0.587 |
|  | [0.257, 0.426] | [0.189, 0.558] | [0.507, 0.763] | [0.391, 0.876] | [3.028, 5.446] | [0.862, 2.495] | [2.671, 4.647] | [0.531, 1.630] | [0.199, 0.347] | [0.324, 1.065] |
| corr X Y | -0.777**** | -0.775**** | -0.925**** | -0.925**** | 0.225 | 0.181 | 0.057 | 0.018 | -0.465** | -0.481* |
|  | [-0.964, -0.591] | [-0.965, -0.586] | [-0.990, -0.860] | [-1.011, -0.839] | [-0.113, 0.564] | [-0.172, 0.534] | [-0.288, 0.401] | [-0.330, 0.367] | [-0.798, -0.132] | [-0.892, -0.071] |
| Age(X) |  | 1.065 |  | 1.152 |  | 0.883 |  | 0.879 |  | 0.966 |
|  |  | [0.906, 1.251] |  | [0.995, 1.335] |  | [0.728, 1.072] |  | [0.692, 1.117] |  | [0.812, 1.151] |
| Age(Y) |  | 0.993 |  | 0.976 |  | 0.555**** |  | 0.461**** |  | 1.521** |
|  |  | [0.822, 1.199] |  | [0.852, 1.119] |  | [0.424, 0.726] |  | [0.340, 0.624] |  | [1.116, 2.073] |
| **Likelihood Ratio Test (p-value)** |  | **n.s.** |  | **n.s.** |  | **p < .00001** |  | **p < .00001** |  | **p < .005** |
| Num.Obs. | 151 | 151 | 157 | 157 | 162 | 162 | 154 | 154 | 156 | 156 |
| AIC | 297.22 | 300.7 | 358.24 | 355.6 | 298.83 | 271.31 | 318.9 | 270.52 | 238.79 | 229.33 |
| BIC | 309.62 | 319.56 | 370.78 | 374.67 | 311.5 | 290.55 | 331.37 | 289.48 | 251.31 | 248.36 |
| * p < 0.05, ** p < 0.01, *** p < 0.001, **** p < .0001 | | | | | | | | | | |

[Bates, D., Mächler, M., Bolker, B., & Walker, S. (2015). Fitting Linear Mixed-Effects Models Using lme4. *Journal of Statistical Software*, *67*(1), 1–48. https://doi.org/doi:10.18637/jss.v067.i01](https://www.zotero.org/google-docs/?j7Pq2Q)

[Kosmidis, I. (2023). *brglm2: Bias Reduction in Generalized Linear Models.* (R package version 0.9.2,) [Computer software].](https://www.zotero.org/google-docs/?j7Pq2Q)

[Kuznetsova, A., Brockhoff, P. B., & Christensen, R. H. B. (2017). **lmerTest** Package: Tests in Linear Mixed Effects Models. *Journal of Statistical Software*, *82*(13). https://doi.org/10.18637/jss.v082.i13](https://www.zotero.org/google-docs/?j7Pq2Q)

[Lenth, R., Singmann, H., Love, J., Buerkner, P., & Herve, M. (2019). *Package ‘emmeans’* [Computer software].](https://www.zotero.org/google-docs/?j7Pq2Q)

[Pinheiro, J. C., & Bates, D. M. (Eds.). (2000). Linear Mixed-Effects Models: Basic Concepts and Examples. In *Mixed-Effects Models in S and S-PLUS* (pp. 3–56). Springer. https://doi.org/10.1007/0-387-22747-4_1](https://www.zotero.org/google-docs/?j7Pq2Q)
